# A therapeutic atlas of monogenic inflammatory bowel disease

**DOI:** 10.1093/ecco-jcc/jjag124

**Published:** 2026-09-12

**Authors:** Pai-Jui Yeh, James E G Charlesworth, Henry Taylor, James J Ashton, Katrina Nash, Kin Hang Lam, Lissy de Ridder, Stephanie A Vuijk, Ziqing Ye, Ying Huang, Tania Bildstein, Wolfram Haller, Kelsey D J Jones, Dror S Shouval, Batia Weiss, Yu Lung Lau, Quynh Bui-Thi-Thuy, Aleixo M Muise, Duncan Richards, Simon Travis, Dan Turner, Holm H Uhlig

**Affiliations:** Centre of Human Genetics, University of Oxford, Oxford, United Kingdom; Translational Gastroenterology and Liver Unit, University of Oxford, Oxford, United Kingdom; Department of Paediatrics, University of Oxford, Oxford, United Kingdom; Division of Pediatric Gastroenterology, Department of Pediatrics, Chang Gung Memorial Hospital, Linkou, Taiwan; Centre of Human Genetics, University of Oxford, Oxford, United Kingdom; Translational Gastroenterology and Liver Unit, University of Oxford, Oxford, United Kingdom; Department of Paediatrics, University of Oxford, Oxford, United Kingdom; Kennedy Institute, University of Oxford, Oxford, United Kingdom; Department of Human Genetics and Genomic Medicine, University of Southampton, Southampton, United Kingdom; Department of Paediatric Gastroenterology, Southampton Children’s Hospital, Southampton, United Kingdom; Oxford Clinical Academic Graduate School, University of Oxford, Oxford, United Kingdom; Oxford University Hospital, Oxford, United Kingdom; Centre of Human Genetics, University of Oxford, Oxford, United Kingdom; Translational Gastroenterology and Liver Unit, University of Oxford, Oxford, United Kingdom; Department of Paediatrics, Willem-Alexander Children’s Hospital/Leiden University Medical Centre, Leiden, The Netherlands; Department of Paediatric Gastroenterology, Erasmus University Medical Centre, Sophia Children’s Hospital, Rotterdam, The Netherlands; Department of Gastroenterology, National Children’s Medical Centre, Children’s Hospital of Fudan University, Shanghai, China; Department of Gastroenterology, National Children’s Medical Centre, Children’s Hospital of Fudan University, Shanghai, China; Paediatric Gastroenterology, Great Ormond Street Hospital for Children, London, United Kingdom; Department of Gastroenterology and Nutrition, Birmingham Children’s Hospital, Birmingham, United Kingdom; Department of Cancer and Genomic Sciences, University of Birmingham, Birmingham, United Kingdom; Paediatric Gastroenterology, Great Ormond Street Hospital for Children, London, United Kingdom; Institute of Gastroenterology, Nutrition and Liver Diseases, Schneider Children’s Medical Center of Israel, Petah Tikva, Israel, affiliated with Gray’s Faculty of Medical and Health Sciences, Tel Aviv University, Tel Aviv, Israel; Pediatric Gastroenterology and Nutrition Unit, Sheba Medical Center, Tel-Hashomer and Gray’s Faculty of Medical and Health Sciences, Tel Aviv University, Tel Aviv, Israel; Department of Paediatrics & Adolescent Medicine, LKS Faculty of Medicine, The University of Hong Kong, Hong Kong SAR, China; Department of Rheumatology, Allergy, and Immunology, Vietnam National Children’s Hospital, Hanoi, Vietnam; Departments of Pediatrics, IMS, and Biochemistry, University of Toronto, Cell and Systems Biology Program, Research Institute, Hospital for Sick Children, Toronto, Canada; Department of Medicine, University of Cambridge, Cambridge, United Kingdom; Translational Gastroenterology and Liver Unit, University of Oxford, Oxford, United Kingdom; Kennedy Institute, University of Oxford, Oxford, United Kingdom; Biomedical Research Centre, University of Oxford, Oxford, United Kingdom; Juleit Keidan Institute of Pediatric Gastroenterology, The Eisenberg R&D Authority, Shaare Zedek Medical Centre, the Hebrew University of Jerusalem, Jerusalem, Israel; Centre of Human Genetics, University of Oxford, Oxford, United Kingdom; Translational Gastroenterology and Liver Unit, University of Oxford, Oxford, United Kingdom; Biomedical Research Centre, University of Oxford, Oxford, United Kingdom

**Keywords:** pediatric inflammatory bowel disease, monogenic inflammatory bowel disease (mIBD), pharmacology, biologics, precision medicine, genomic medicine

## Abstract

**Background and aims:**

Evidence-based, mechanism-guided therapies are urgently needed for treating monogenic inflammatory bowel disease (mIBD). For such rare diseases, mechanistic insight is essential to guide treatment when conventional clinical trials are often not feasible. We aimed to summarize literature-based evidence and to identify knowledge gaps.

**Methods:**

We conducted a systematic review of published manuscripts evaluating the therapeutic efficacy in mIBD. We quantified and compared the global therapeutic response score across treatments and conditions. In a subset of conditions, biomarkers of longitudinal therapeutic response were evaluated in comparison to non-monogenic pediatric IBD cohorts.

**Results:**

Responses to 35 therapeutics across the 102 known genetic causes of mIBD were evaluated in 241 articles and 669 patients, summarizing 302 gene–drug responses. The efficacy of at least one pharmacological intervention was identified in 61% (*n* = 62/102) of the mIBD conditions, highlighting a major unmet need for effective medications in many others. Gene- and pathway-specific responses were demonstrated for several therapies, including allogeneic hematopoietic stem cell transplantation, gene therapy, and advanced therapies such as anti-TNF agents, IL-1 inhibitors, mTOR inhibitors, as well as eculizumab in CD55 deficiency, abatacept in CTLA4 deficiency, and the immunometabolic agent empagliflozin in glycogen storage disease type 1b.

**Conclusions:**

This study highlights the potential of precision medicine approaches tailored to genetic and pathway-specific mechanisms, while underscoring the urgent need for effective therapies in many monogenic conditions that remain without established treatment options.

## Graphical Abstract

**Graphical Abstract:**
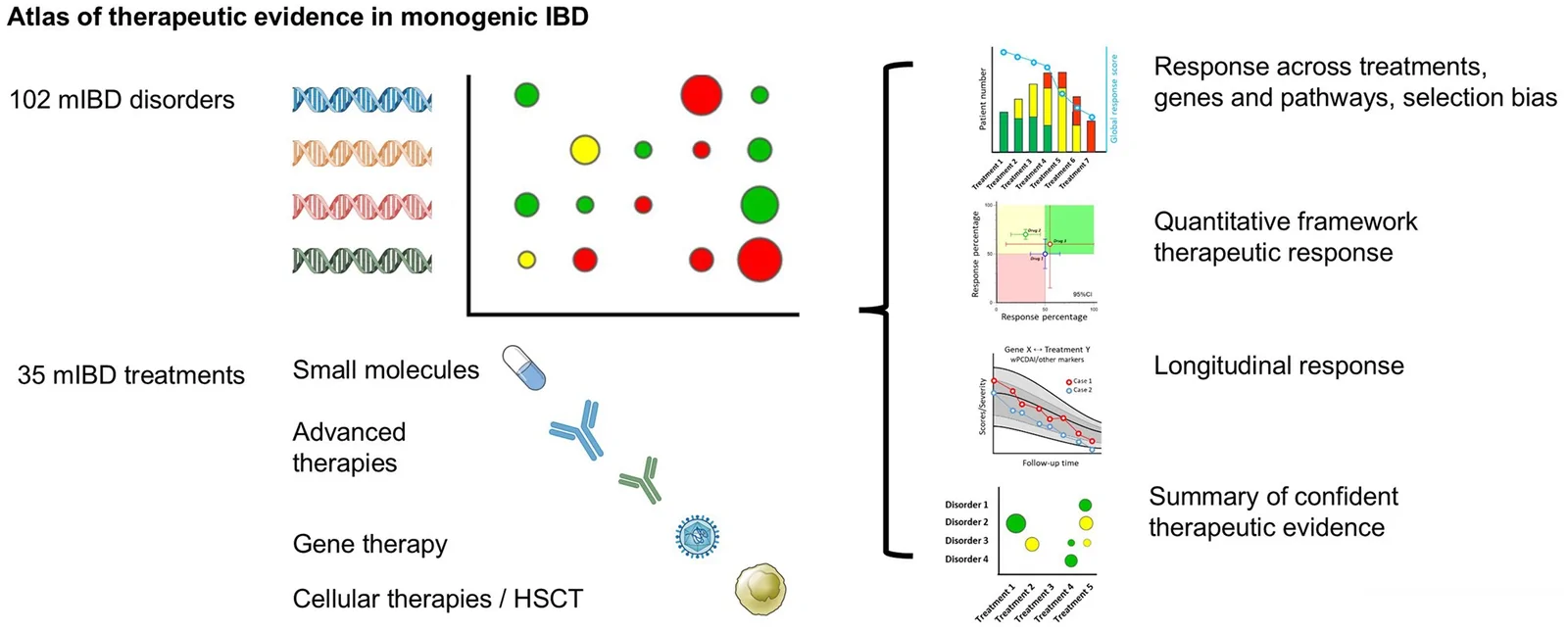

## 1. Introduction

Understanding the genetic basis of disease can inform target identification and support the development of pathway-specific therapies.^1^ This is especially true for rare genetic disorders, where highly penetrant genetic variants cause significant functional perturbation, resulting in severe disease. While Mendelian disorders are potentially less complex than polygenic disorders, the translation from causative mechanisms to evidence-based treatment is challenging.

Inflammatory bowel disease (IBD) results from a pathophysiologic process that combines genetic susceptibility and environmental exposures, driving microbiome imbalance, mucosal barrier defects, and defective immune homeostasis.^2^ IBDs typically have a polygenic basis, where the aggregated risk from hundreds of alleles contributes to the risk of the development of intestinal inflammation.^3^ A distinct subgroup of patients has monogenic IBD (mIBD), caused by pathogenic variants in single genes that disrupt mucosal barrier integrity or intestinal immune homeostasis.^4^ Over 100 monogenic disorders have been identified that cause intestinal inflammation with moderate to high penetrance.^5^ While patients with mIBD are often endoscopically and histologically indistinguishable from those with polygenic IBD, they tend to present early in life, with a large proportion developing intestinal inflammation in infancy.^6^ They may also have additional congenital anomalies, susceptibility to infection, inborn errors of immunity, severe systemic autoimmunity, and early-onset malignancy.^7^

Patients with mIBD are insufficiently served by existing treatments. Although a wide range of therapeutics are theoretically available for IBD, there is a lack of gene variant-specific evidence for induction or remission of intestinal inflammation, and most therapeutics are not approved for use in young children—none for those under 6 years of age—or for IBD specifically.^8^ Many of these broad-brush immune modulators may not be relevant for mIBD mechanisms, leading to ineffective treatment regimens, high disease burden, healthcare utilization, and additional risks for patients. Mechanistically informed approaches would enable the right patient to receive the proper treatment at the right time.^9^

The established molecular and genetic basis in some patients with mIBD may guide pathway-directed medications, earlier hematopoietic stem cell transplantation, or gene therapy.^10^ However, traditional randomized controlled trials are impractical and unrealistic for most specific genetic conditions underlying mIBD. In the absence of prospective trial data, a rational and pragmatic approach is a structured investigation of retrospective evidence to gain real-world insights from clinical experience. This enables an overview of reported therapeutic responses in the different types of mIBD. We systematically reviewed treatments reported to have been used in mIBD and identified medications with promising therapeutic efficacy for specific pathways.

## 2. Materials and methods

### 2.1. Structured literature review and search strategies

Eligible studies were identified via a systematic literature review. MEDLINE and PubMed Central databases were searched for full-text articles published in English between January 1, 2000 and April 4, 2023. Literature before 2000 was excluded as the standard of care has changed, and genetic information during that period is limited, since most mIBD genes were discovered after 2009. Databases were searched for the keywords “inflammatory bowel disease (inclusive of ‘ulcerative colitis’ and ‘Crohn’s disease’),” the gene name, and the specific treatment, combined with synonyms and Medical Subject Headings (MESH) terms, along with the relevant condition. We also reviewed all previously identified publications of mIBD cases, collated by Bolton et al.^5^ Publications were screened to exclude duplicate reports of patients (using detailed case descriptions, genetics, authors, and their affiliations). Where a patient was included in multiple publications, preference was given to the article that provided the most detailed information on IBD outcomes. However, if outcomes were revised in later publications, these revisions took precedence. Abstracts underwent primary assessment for inclusion. Additional cases were identified by screening references, associated supplementary files, and citations.

Eight independent researchers participated in the paper review process (material identification and primary assessment), using a standardized reporting worksheet for data extraction. Two researchers independently reviewed each manuscript and coordinated data collection before data integration. Discrepancies in the assessment between reviewers regarding inclusion were resolved by consensus after discussion with the entire team. The eight researchers are medically qualified for assessing the therapeutic response: pediatric gastroenterologists (PJY, ZY, TB, JJA), a clinical lecturer in pediatric infectious disease and immunology (JEGC), a medical registrar in pediatric gastroenterology (HT), an academic foundation trainee in pediatric gastroenterology (KN), and a medical student (KHL).

### 2.2. Inclusion criteria

Eligible cases fulfilled the following criteria. (1) A diagnosis of IBD or inflammatory enteropathy, identified using keywords of IBD, Crohn’s disease (CD), ulcerative colitis (UC), IBD unclassified (IBD-U), enteropathy, and other synonyms within a reasonable context (colitis, ileitis, enteritis). The diagnostic evidence needed to be consistent with clinical, endoscopic, and histopathological examinations as outlined in the revised Porto guidelines.^11^ Cases with a likely alternative cause of the intestinal inflammation, such as infection, graft versus host disease, or medication-induced colitis, were excluded. (2) A confirmed genetic diagnosis was required, specifying the gene name and relevant variant information (eg, pathogenic DNA or protein consequence). When available, functional assays were used to support the classification of likely pathogenic variants. (3) A treatment with one of 35 selected therapies as defined below (including cellular, biologic, and small molecules), and a record of the therapeutic response of intestinal inflammation (even if the treatment was primarily toward extraintestinal manifestations, such as susceptibility to infection with inborn errors of immunity).

### 2.3. mIBD genes

The mIBD panel of this study included 102 genes previously described as having moderate to high penetrance and demonstrable disease-causing potential.^5^ For genes in which variants drive different clinical syndromes or pathotypes, such as gain or loss of function (GOF or LOF) variants, respectively, these were grouped and analyzed separately.

### 2.4. Treatment modalities

The 35 selected treatments were derived from an extensive literature review on mIBD therapeutics,^9^ and grouped by five major categories reflecting different therapeutic mechanisms. (1) Small molecules: mTOR inhibitor (sirolimus), ubiquitinylation (thalidomide), calcineurin phosphatase inhibitor (tacrolimus), JAK inhibitor (tofacitinib, ruxolitinib, baricitinib), inosine monophosphate dehydrogenase inhibitor (mycophenolate), block of microtubule assembly (colchicine), angiotensin receptor 1 inhibitor (losartan), sodium glucose co-transporter 2 inhibitor (empagliflozin, dapagliflozin), rescue of glycosylation defect (mannose or fucose). (2) Biologics (anti-TNF, recombinant TNF receptor, anti-A4b7 integrin, anti-IL-1b, IL-1 receptor antagonist, CTLA-4 fusion protein, recombinant IL-18 binding protein, anti-CD20, anti-IL-12/23p40, anti-IL-6 receptor, anti-CD25, anti-complement 5, and anti-TNF-non-specified [where reports did not specify]). (3) Growth factors (G-CSF growth factor) and intravenous immunoglobulin (IVIG) replacement therapy: these drugs were recorded only if there was sufficient information to assess the effect on intestinal inflammation (while licensed for the underlying immunological or hematopoietic disorder). (4) Cellular therapy: allogenic bone marrow, peripheral blood or umbilical cord blood hematopoietic stem cell transplant (alloHSCT, alloUCB-HSCT). (5) Gene therapy (retroviral transfer, lentiviral transfer).

The efficacy of standard of care treatments such as aminosalicylates, corticosteroids, thiopurines, or exclusive enteral nutrition were not analyzed as clinical responses to these therapies were not systematically reported. Poor response of mIBD to standard of care therapies and/or frequent need for escalation to advanced therapies has been reported previously.^8^ We aimed to focus on advanced therapeutic agents used for maintenance of remission, rather than induction alone.

### 2.5. Global treatment response

Responses to therapy was stratified into three groups as reported in the original publications: (1) response (R), defined as resolution of inflammation and no need for subsequent drug escalation—supported by biochemical, macroscopic, or histological mucosal healing; (2) partial response (PR) indicated by improved condition but residual inflammation with a need for further drug adjustment (therapy escalation; add-on or therapy); and (3) no response (NR)—lack of primary efficacy or primary drug intolerance leading to withdrawal.

A weighted mean global therapeutic response score (GTRS) was calculated to average individual responses (non-response, partial response, and response, weighed as 0, 1, and 2, respectively). The relative efficacy of individual treatments was visualized (five-scale colors to divide the GTRS) against case numbers (symbol size stratified by <5, 5-10, 11-20, 20-50, and >50 cases).

Confidence intervals for three-scale semiquantitative responses (R/PR/NR) are difficult to calculate due to a lack of meaningful scaling.^12^ These categories are non-linear and might differ across mIBD disorders; statistical methods based on mean therapeutic scores and their confidence margins are lacking. We therefore defined the proportion of patients with a complete response as a relevant outcome. This incorporates patient numbers and therapeutic effect size by condition, allowing for aggregation by biologically functional or phenotypic sub-group (eg, IL-10 defects) or by merging classes of therapeutics (eg, anti-TNF) that show efficacy range by condition. Through integrating the confidence intervals of complete response (percentage of patients achieving response, R) and at least partial response (percentage of patients achieving partial or complete response [PR + R]), the therapeutic efficacy of an intervention for a particular condition can be compared. Confidence intervals were calculated based on the modified Wald equation.^13^ In the confidence plot analysis, we defined a therapy as having “moderate therapeutic evidence” if >50% of patients had partial (PR) or complete response (R), or “strong therapeutic evidence” if >50% of patients had a complete response (R).

### 2.6. Benchmark comparison to treatment in paediatric Crohn’s disease

To compare the clinical responses of anti-TNF therapy in patients with mIBD to those with classical IBD, we assessed data from a study investigating the longitudinal response of anti-TNF agents (infliximab) in pediatric CD patients. We examined six outcome parameters based on an open-label multicenter randomized controlled trial (assessed at week 0, 6, 10, 14, 22, and 52; patients received five infusions of infliximab and continued on azathioprine monotherapy, unless the patient was not in remission at 22 weeks) of 49 infliximab-treated children who were newly diagnosed with moderate-to-severe CD.^14^ These parameters included white blood cell count (WBC), erythrocyte sedimentation rate (ESR), C-reactive protein (CRP), fecal calprotectin (Fcp), and the weighted pediatric Crohn’s disease activity index (wPCDAI).

### 2.7. Novel case series

Therapeutic histories and treatment responses to IL12/23(p40) targeting ustekinumab, selective anti-IL-23 agents (p19 inhibitors), or anakinra were obtained through personal communication from five unpublished patients with IL-10 receptor defects and IBD, a more common form of mIBD (Supplementary data).

### 2.8. Statistical analysis and visualization

Dimensionality reduction was performed based on ClustViz. Data analysis and visualization were performed using GraphPad Prism 10 software (GraphPad software, Inc., San Diego, CA, USA).

### 2.9. Ethical statement

This study type is exempt from IRB review as ethical approval is not required for the review of published literature. We include five previously unpublished cases of IL-10 receptor defects, where written informed consent was obtained from these patients or their parents/guardians for publication.

## 3. Results

### 3.1. Assessment of published therapeutic evidence in monogenic IBD

In total, 1572 manuscripts were screened, of which 241 were included in the analysis, summarizing 969 patient reports altogether. This comprises 96 single-case reports (40%), 69 single-center case series (29%), 56 multi-center case series (23%), three population/registry studies (1.2%), four clinical trial studies (1.7%), and 13 reviews or systematic reviews (5.4%). Almost all were retrospective observational studies (98%). None of the included papers reported on a complete set of standardized parameters as suggested by the mIBD REPORT criteria.^9^^,^^15^

**Figure 1.**
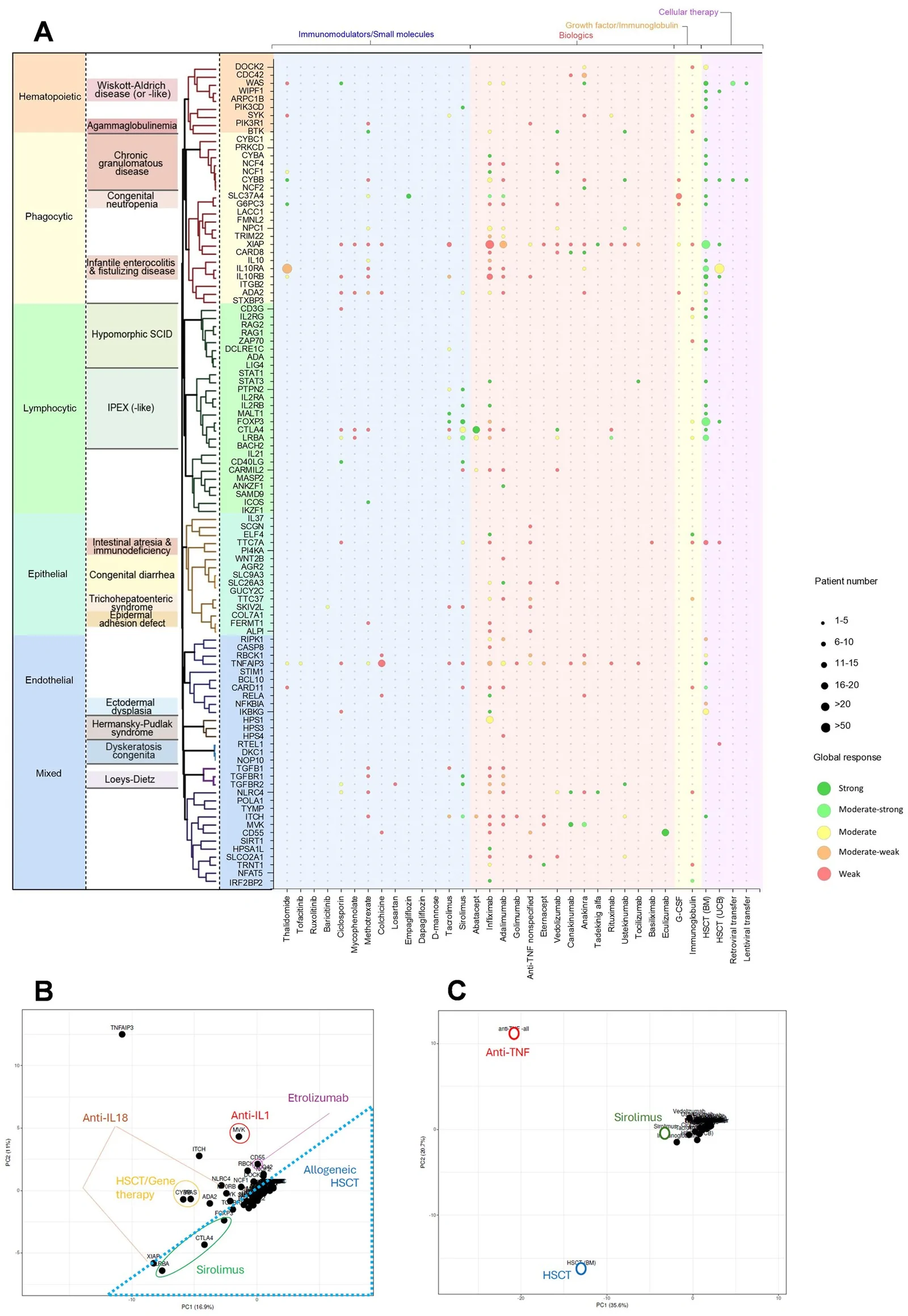
Pharmacological atlas of mIBD. (A) Efficacies of 35 therapeutics were assessed in 102 mIBD conditions and presented as global therapeutic response score (GTRS). Conditions are arranged according to previously published functional groups.^5^ (B) Principal component analysis highlights groups of genes and treatments that drive the components (frequency of reporting). (C) Principal component analysis of treatments highlights the contribution of anti-TNF, allogeneic HSCT, and sirolimus.

A specified intervention outcome was reported for 73 of 102 (72%) mIBD conditions, and for 33 of 35 (94%) of the included therapies. The published data demonstrated 302 gene–drug–response interactions, but the strength of evidence is limited since 264/302 (87%) interactions included fewer than five reported cases (Figure 1A; Table S1).

### 3.2. A therapeutic atlas for mIBD

**Figure 2.**
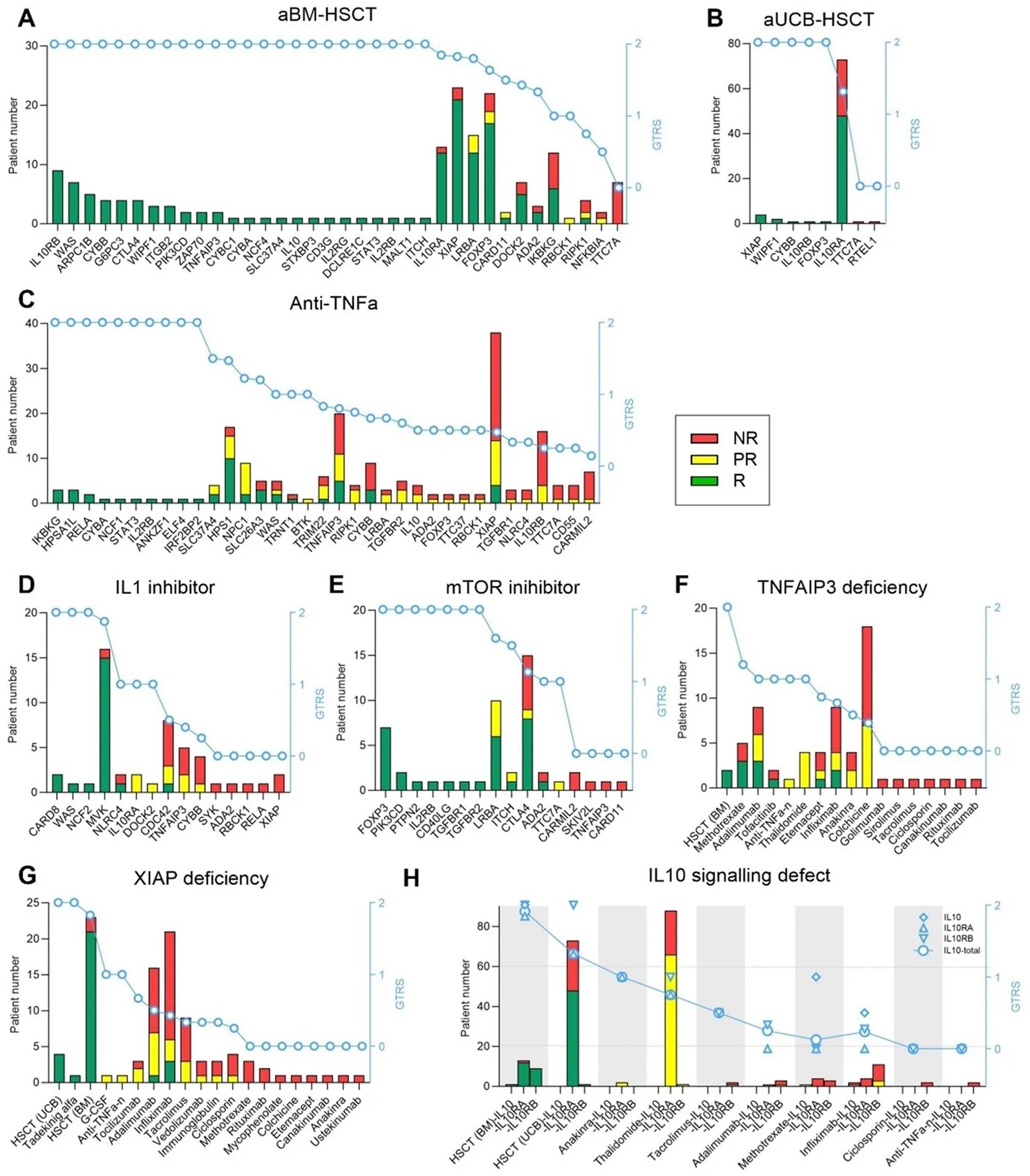
Comparative analysis of global therapeutic response scores across genes and treatments. (A–E) Global therapeutic response score of treatments: (A) allogeneic BM-HSCT, (B) allogeneic-UCB-HSCT, (C) anti-TNF, (D) IL-1 inhibitor, and (E) mTOR inhibitor); and (F–H) across conditions: (F) TNFAIP3 haploinsufficiency, (G) XIAP deficiency, (H) IL-10 signaling defects (*IL10RA*, *IL10B*, and *IL10*).

Among the 35 therapeutic interventions, anti-TNF was the most frequently reported in 53 conditions (52%), followed by allogenic HSCT in 37 conditions (36%) (Figure 1A). Multiple interventions were reported for some conditions, including XIAP deficiency (*n* = 20 interventions), TNFAIP3 (A20 haploinsufficiency, *n* = 17), CYBB (X-linked chronic granulomatous disease, *n* = 11), and CTLA4 haploinsufficiency (*n* = 11) (Figure 2A). Dimensionality reduction analysis of treatment data across conditions highlights key treatments, including allogeneic stem cell transplantation, anti-TNF therapy, and mTOR inhibitors (Figure 1B and C).

### 3.3. Cellular and pharmacological therapies

Indication for effective therapeutic response to allogeneic HSCT (GTRS > 1) was observed across 31 mIBD genes (Figures 1A and 2A, B). Allogenic HSCT had greater efficacy in mIBD conditions caused by variants affecting the hematopoietic compartments (particularly phagocytes and lymphocytes, Figure 1A). A substantial amount of data were reported for patients with XIAP, FOXP3, and IL-10 defects. Meanwhile, HSCT has shown limited efficacy in disorders involving epithelial cell defects, such as those caused by TTC7A mutations.

The effectiveness of anti-TNF treatment was variable across various monogenic conditions (Figures 1A and 2C), with the clearest therapeutic evidence in conditions with phagocyte defects, including CGD, congenital neutropenia, Niemann Pick type C, and Hermansky–Pudlak syndrome type 1 (*HPS1*) (Figures 1A and 2C). A similar heterogeneity of therapeutic effectiveness was also noted in IL-1-targeting treatments (anakinra, canakinumab) (Figure 2D).

In immunodysregulation polyendocrinopathy enteropathy X-linked syndrome (IPEX syndrome) or IPEX-like conditions, in addition to allogeneic HSCT, we observed some degree of therapeutic effectiveness among treatments with diverse mechanisms of action, such as sirolimus (Figure 2E**)**, tacrolimus, CTLA4-fusion protein (abatacept), and anti-TNF. For TNFAIP3 variants (A20 haploinsufficiency), available data suggest the effectiveness of allogeneic HSCT, while no other treatments are likely comparable (Figure 2F). Data in XIAP deficiency highlight a list of interventions with unsatisfactory effectiveness, except for allogeneic HSCT (Figure 2G).

Mechanistically guided precision medicine is indicated in a number of diseases (Figure 1A). Gene therapy benefited patients with Wiskott–Aldrich syndrome (WAS) and chronic granulomatous disease (CGD) due to pathogenic *CYBB* variants. Empagliflozin, a sodium-glucose co-transporter 2 (SGLT2) inhibitor, exhibited a strong therapeutic response in glycogen storage disease type 1b (GSD1b), which is caused by an SLC37A4 variant. IBD caused by C55 deficiency, leading to dysregulation of the complement cascade, was effectively treated with eculizumab, which inhibits the cleavage of the C5 complement protein.^16–21^ Patients with mevalonate kinase deficiency, which results in IL-1β overproduction, failed to respond to anti-TNF biologics (infliximab, adalimumab, and etanercept) but had a good response to anti-IL1 (anakinra or canakinumab).^22–26^

### 3.4. Medications with overlapping targets and drug mechanisms induce similar responses

We hypothesized that medications with the same mechanism of action are likely to exhibit a similar therapeutic response across different conditions with the same pathway defects. Indeed, anti-TNF therapies (infliximab and adalimumab) as well as IL-1-targeting therapies (IL-1R antagonist anakinra and anti-IL-1β canakinumab) demonstrated similar GTRS across diseases within several conditions (Figure 1A). This suggests that merged outcome data enhance confidence for individual conditions.

### 3.5. Genetic conditions with shared pathways have similar therapeutic outcomes

We analyzed the therapeutic response of genetic conditions with defects in similar molecular pathways. This suggests that gene defects sharing functional mechanisms and phenotypes were successfully treated by medications with a similar mechanism of action.

For instance, several disorders characterized by defective regulatory T cell function (eg, *FOXP3*, *IL2R*, *PTPN2*, *CTLA4*, *LRBA*) responded well (strong-to moderate efficacy) to the mTOR inhibitor sirolimus (Figure 2E). Both CTLA4 and LRBA (a CTLA4 binding protein) deficiencies showed a good therapeutic response to the CTLA4 fusion protein abatacept (Figure 1A). Similarly, IL-10 signaling defects (deficiencies of *IL10RB*, *IL10RA*, or *IL10*) resulted in consistent responses across differing treatment modalities (Figure 2H).

This provides evidence that in genetic conditions with closely shared pathways and clinical phenotypes, treatment responses might be extrapolated.

### 3.6. Indication for publication bias toward a positive therapeutic outcome

There was an indication of strong publication bias toward positive therapeutic reports in genes with small case series, overrepresenting positive findings, while larger cohorts reflected a more nuanced outcome (highlighted in allogeneic HSCT and anti-TNF, Figure 2A and B).

To assess the degree of publication bias of missing negative data, we conducted a selective survey reaching toward international pediatric gastroenterology centers. We focussed on the group of IL-10 signaling defects that are refractory to anti-TNF agents, where patients were likely treated with alternative advanced therapies. We recorded five clinical cases with IL-10 signaling defects (three patients with *IL10RB* variants and two with *IL10RA* variants) who have been treated with ustekinumab or anakinra *without* being reported because of partial or non-response (Supplementary data—Case Reports). Although these unpublished reports do not demonstrate a lack of efficacy of anti-IL-12/-23 or IL-1R antagonist in IL-10 receptor deficiencies, they do highlight an important publication bias and the need for systematic analysis beyond published data.

### 3.7. Comparison between global therapeutic response and quantitative therapeutic response modeling based on the mIBD report standards

Whilst these treatment data represent the most pragmatic best available published evidence, there are clear limitations in using a reductive global therapeutic response score for treatment outcome. To validate the GTRS as a proxy, we compared its performance relative to quantitative data captured using the recently described mIBD reporting standards. ^9^

**Figure 3.**
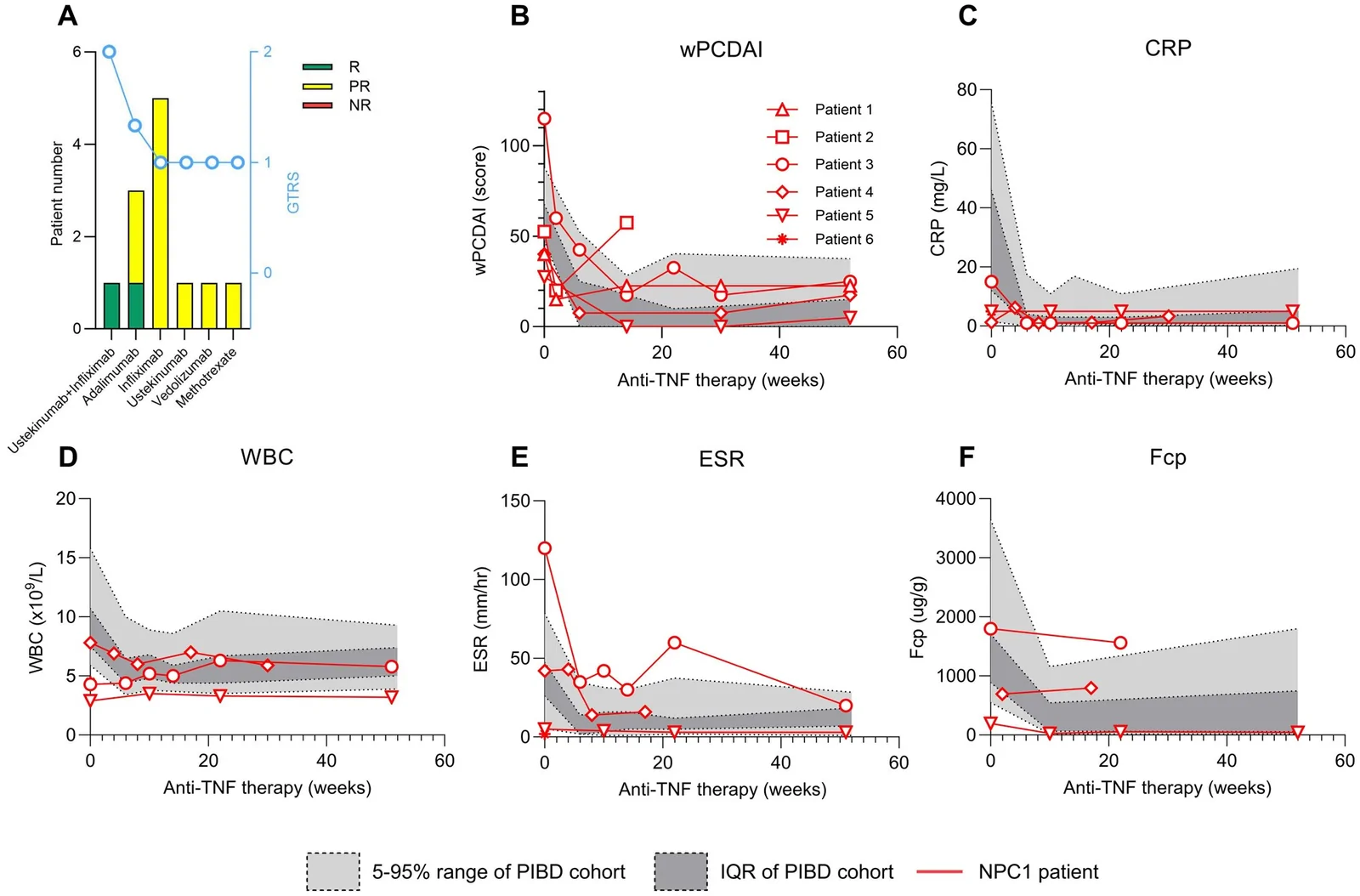
The therapeutic response to anti-TNF therapy in patients with NPC1 mIBD compared with classical pediatric Crohn’s disease. (A) Comparative analysis of different treatments. (B–F) Longitudinal response to anti-TNF therapy illustrated by (B) weighted Crohn’s disease activity index (wPCDAI), (C) C-reactive protein (CRP), (D) white blood count (WBC), (E) fecal calprotectin (Fcp), and (F) erythrocyte sedimentation rate (ESR). The response of patients with pediatric Crohn’s disease is shown as a benchmark comparison. The dark gray area illustrates the interquartile range, while the light gray area represents the 5%-95% range of a cohort of pediatric patients (*n* = 49).

We utilized data from a cohort of patients with Niemann–Pick disease type C1 (NPC1), a disorder associated with CD-like immunopathology, where increased TNF is observed, and anti-TNF treatment has been reported to be effective.^27^^,^^28^ The GTRS suggested a moderate response (adalimumab GTRS 1.3; infliximab GTRS 1.0) (Figures 2C and 3A).

Use of the more granular mIBD REPORT criteria (Supplementary data) confirmed this partial response. After 52 weeks of treatment, an improvement (an average of >60% reduction of scores) in the wPCDAI was observed in 80% of patients (Figure 3B-F). Therapeutic responses in these biologic-naive NPC patients were benchmarked against a cohort of biologic-naive children with classical pediatric CD (pIBD) treated with infliximab. (Figure 3B-F, background grey areas). This demonstrates that anti-TNF treatment in patients with NPC1 provides partial but not complete efficacy, although current data are insufficiently powered to determine whether responses are comparable to or inferior to those seen in classical pIBD.

The data overall suggest that, in the absence of prospective granular outcome markers, the GTRS serves as a reasonable surrogate to evaluate confidence in responses to reported therapy.

### 3.8. A quantitative framework identifies high-confidence therapeutic interventions

We sought to describe the therapeutic response using a statistical framework that measures the percentage of patients with a complete or at least partial response and provides confidence intervals.

**Figure 4.**
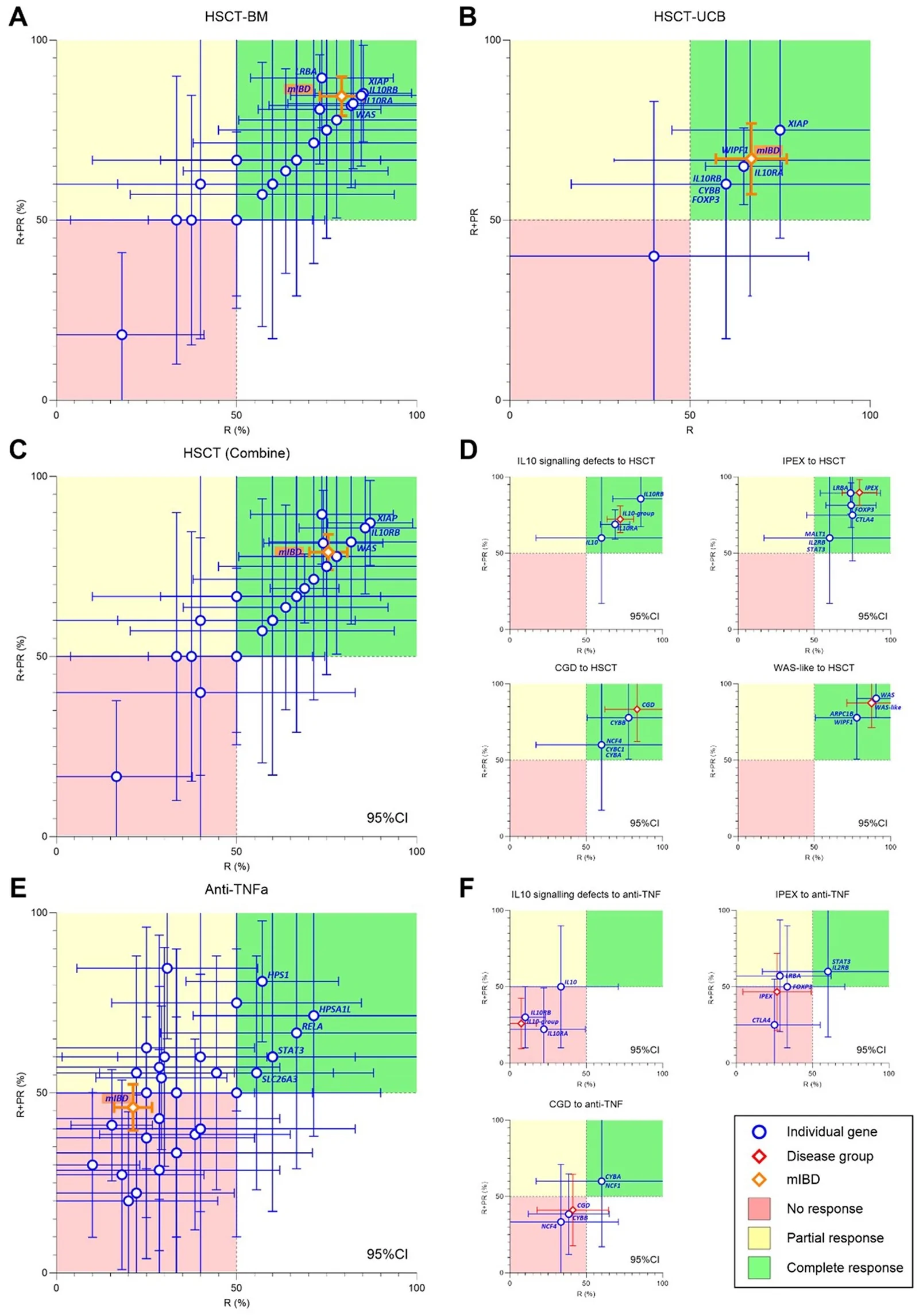
A quantitative framework of therapeutic response in mIBD. Percentage of mIBD patients with complete and partial therapeutic responses, implementing 95% confidence intervals. (A) Allogenic HSCT-associated treatment response across multiple mIBD conditions. (B) Umbilical cord blood HSCT-associated treatment response across multiple mIBD conditions. (C) Allogenic HSCT (combining A and B)-associated treatment response across multiple mIBD conditions. (D) Individual and disease group response to allogenic HSCT among four mIBD disease groups. (E) Anti-TNF-associated response across mIBD conditions. (F) Individual and disease group response after anti-TNF among the three mIBD disease groups.

This is exemplified for the most common treatments, allogeneic HSCT (Figure 4A-D) and anti-TNF agents (Figure 4E and F) across diseases. Outcomes among four disorder groups (IL-10 signaling defects, IPEX, CGD, and WAS-like disease, Figure 4D) support disease-specific interpretation while strengthening confidence when evidence is merged at the disease group rather than gene level. In comparison, a generally poor response to anti-TNF agents was observed across many disorders (Figure 4E and F), whereas certain individual disorders demonstrated partial therapeutic efficacy (Figure S1).

**Figure 5.**
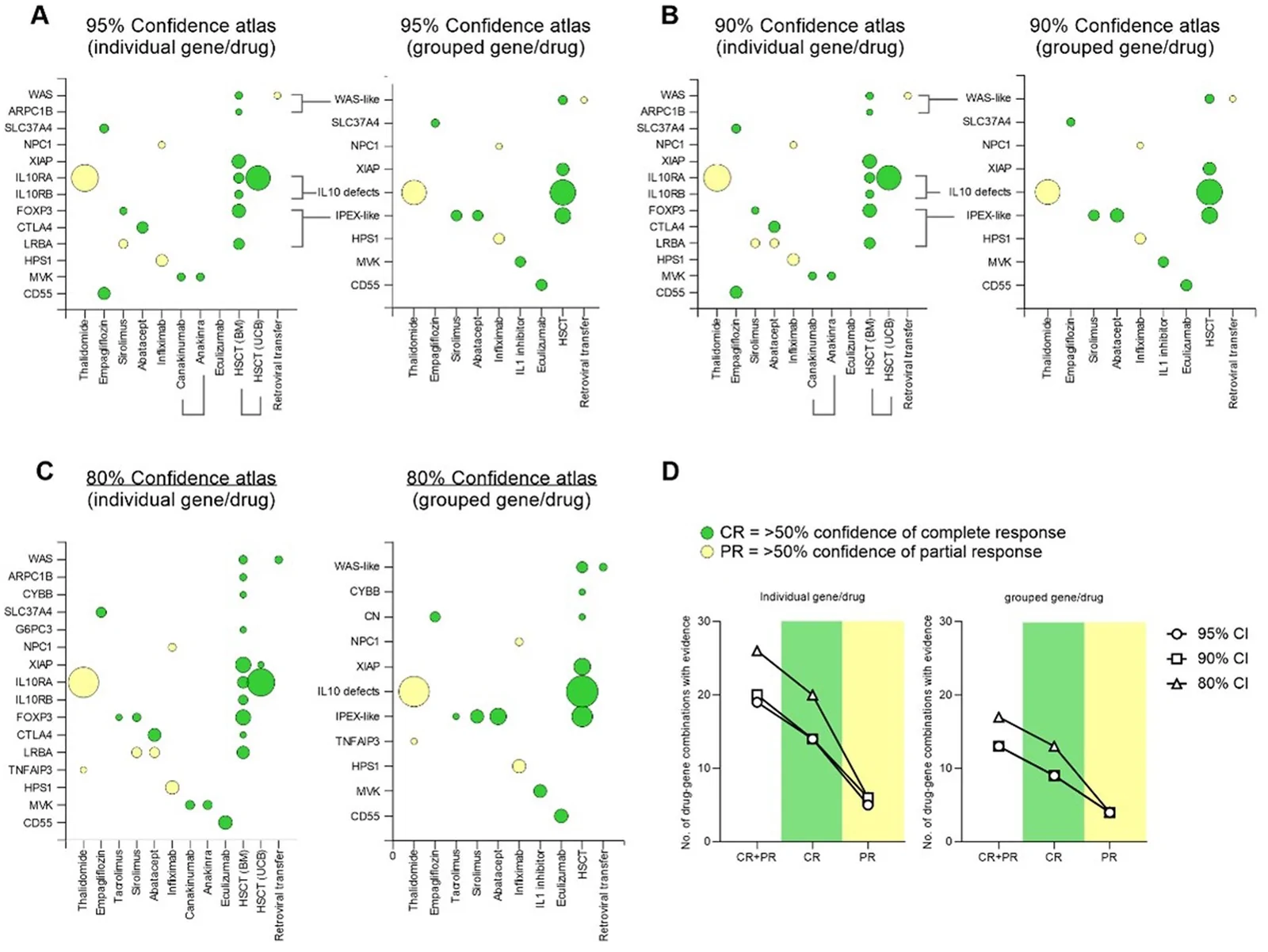
From treatment reports to confidence therapeutic atlas. From nominal drug–gene combinations towards a map of high confidence of therapeutic responses. Complete response (green) defines >50% of patients of the condition with high confidence of complete response; partial response (yellow) defines >50% of patients of the condition with high confidence of complete or partial response. (A) In addition to the individual gene/drug 95% confidence atlas covering individual drug–gene combination, the grouped gene/drug atlas shows merged disease groups or drug mechanisms. (B & C) Two different levels of confidence interval (90%, 80%). (D) Comparison of the number of drug–gene combinations covered by different confidence levels.

Based on the concept of therapeutic confidence, we filtered the primary atlas to select drug–gene combinations with stronger evidence (at least with moderate therapeutic evidence) (Figure 5). This condenses the 302 sets of drug–gene combinations to 19 with high confidence of therapeutic response. This can be distilled into 13 drug–pathway combinations by merging biological phenotypes (eg, IL-10 signaling) and drug classes (eg, anti-TNF or IL-1 antagonists). Although the number of effective combinations is reduced, there is higher confidence in the remaining classes of disorders and genes, providing greater value for clinical translation.

While therapeutic responses with high confidence (90% and 95%) are preferred for identifying therapeutics with the best evidence (Figure 5A and B), using a lower confidence threshold (such as 80%) is crucial for identifying a focus for immediate research opportunities (Figure 5C).

Overall, this approach indicates the feasibility of using a clinically meaningful semi-quantitative assessment of therapeutic response based on statistical confidence (Figure 5D).

## 4. Discussion

We present a systematic analysis of therapeutic outcomes of 35 therapeutics across the 102 known genetic causes of mIBD. The atlas supports the concept of mechanism-based therapies, elucidates the evidence gap in mIBD, and highlights areas for future research. We demonstrate comparable therapeutic responses across genetic conditions with shared disease mechanisms, highlight the potential of precision medicine approaches in selected disorders (including gene therapy, allogenic HSCT, and IL-1-targeted therapies), and underscore the unmet need in many conditions that currently lack effective treatments. Perhaps unsurprisingly, we identify evidence of publication bias favoring the reporting of positive outcomes.

The value of allogeneic HSCT is underscored by its high effectiveness in disorders involving hematopoietic cell line dysfunction, while recognizing its limited role in conditions such as TTC7A or other epithelial defects. Strong therapeutic responses have also been observed with eculizumab in CD55 deficiency, IL-1-targeted therapies in MVK deficiency, and empagliflozin in GSD1b caused by pathogenic variants in *SLC37A4*. The utility of pathway-specific therapy is further exemplified by the comparable efficacy of anti-TNF agents (infliximab and adalimumab) across disorders with shared pathway defects, as well as the benefit of IL-1 blockade (anakinra and canakinumab) in MVK deficiency. Collectively, these examples highlight the potential of precision medicine strategies that directly target disease pathophysiology. This supports the concept that drug mechanisms supported by genetic evidence are more likely to succeed.^1^

The implementation of novel treatments for mIBD relies largely on a clear understanding of disease pathophysiology, the development of new therapeutic approaches, or the availability of authorized medications that can be repurposed. When there is a strong biological rationale and well-documented initial cases demonstrating substantial therapeutic benefit, the adoption of such therapies as standard of care in monogenic conditions can occur rapidly. A notable example is the treatment of patients with GSD1b who present with neutropenia and intestinal inflammation, where clinical translation to practice occurred within 5 years of pathway identification. The use of the SGLT2 inhibitor empagliflozin in GSD1b has been reported in both pediatric and adult patients, demonstrating normalization of neutropenia and neutrophil dysfunction, with unexpected concurrent improvements in mucosal lesions and CD-like intestinal inflammation.^29^

In addition to this primary atlas describing the nominal landscape across a wide spectrum of responses, our statistical assessment provides evidence for treatment effects. This approach also identifies promising candidates for future translational research. Based on specific genetic defects and affected functional pathways, emerging drugs targeting key disease mechanisms can be anticipated for implementation in the mIBD field. These include IL-2 or IL-10 signaling, prostaglandin metabolism, JAK/STAT inhibitors (eg, upadacitinib and filgotinib, both targeting JAK1),^30^ phosphodiesterase 4 (PDE4) inhibitors,^31^ SYK inhibitors,^32^ new C5 inhibitor (pozelimab),^33^ and IL-18-targeting biologics.^34^ Novel therapeutic concepts include gene therapy (ie, targeting FOXP3, NCF1, and ADA2),^35–37^  *in vitro* expanded Treg cell therapy or *in vivo* Treg expansion with IL-2 stimulation, enhanced IL-10 signaling via IL-10 fusion proteins (IL-10/anti-CD86 fusion protein [APVO210, ADAPTIR], IL-4/IL-10 fusion protein, cetuximab),^38^^,^^39^ oral short chain fatty acids such as butyrate,^40^ sodium-glucose co-transporter-2 (SGLT-2) inhibitors, or leflunomide in TTC7A deficiency.^41^ The diversity is daunting and underscores the need to quantify reports of response. Knowing which treatments cannot be expected to work is important for clinical decision-making, since this saves patients from inappropriate intervention.

In this study, we also describe essential knowledge gaps in the current literature. For more than 80% of interventions, outcome data are derived from fewer than five reported cases. Moreover, for approximately 30% of mIBD conditions, no intervention targeting gastrointestinal disease has been reported at all.

The study is limited by heterogeneity in reporting, including differences in clinical standards, healthcare systems, outcome measures, and definitions of response across institutes and countries. We acknowledge that a degree of interpretive bias could exist despite best possible practice of different reviewers and resolution of discrepancy discussions. In addition, we have highlighted that this study is also limited by publication bias, which may lead to selective reporting and a potential overrepresentation of patients with more severe phenotypes within the clinical spectrum. Indeed, we found that single case reports describing novel treatment strategies were more likely to report positive outcomes, while larger series (single- or multicenter cohorts) provided more nuanced data. However, any clinician attempting to interpret the current literature in order to provide balanced advice to patients faces the same underlying limitations and potential biases. In contrast, our atlas offers a systematic, comparative, and quantitative assessment of the available evidence. Significant research is required in this field to recognize the long-term therapeutic efficacy, monitor adverse events, compare real-world evidence prospectively, and establish gold-standard randomized clinical trials where appropriate.

Establishing a standardized prospective reporting system is a promising strategy to generate a less biased, dynamic, and comprehensive database of mIBD therapeutic efficacies as we advance. With standardized reporting, clinicians caring for those with mIBD could register anonymized cases and report essential patient information within a unified structure, including a reliable genetic diagnosis, phenotypic classifications, treatments, and outcomes or adverse events.^15^

This analysis supports the concept that genetic conditions with shared disease mechanisms can exhibit similar responses to therapeutic agents. It emphasizes the promise of precision medicine in selected disorders, while underscoring the urgent need for effective therapies in many conditions where they are lacking. At the same time, evidence of selection bias—favoring the reporting of positive over negative outcomes—highlights important limitations within the existing literature. Together, these insights offer an initial framework for guiding the development and evaluation of precision therapies.

## Supplementary Material

jjag124_Supplementary_Data

## Data Availability

The authors confirm that the data supporting the findings of this systematic review are available within the article and its supplementary materials. Limited, anonymized, and explicitly consented, data from the five patient case reports may be made available on reasonable request to the corresponding author, H.H.U. All other data that support the findings of this study are available from the corresponding author, H.H.U., upon reasonable request.
