# Supplementary material for: A therapeutic atlas of monogenic inflammatory bowel disease
Source: J Crohns Colitis. 2026 Sep 12;20(9):jjag124. doi: 10.1093/ecco-jcc/jjag124 (PMC13570588; doi:10.1093/ecco-jcc/jjag124)
Supplement: Supplementary file 1 [file jjag124_supplementary_data.zip › Supplemental file - mIBD atlas- 08092026-clean.pdf]

Title: A therapeutic atlas of monogenic inflammatory bowel disease

Supplemental data

Contents

Supplementary Table 1. Summary of included articles by gene defect 2

Supplementary Figure 1. Quantified therapeutic response ranges for therapeutics used in mIBD. 5

Supplementary case reports: Case history of patients with IL10 defects treated with ustekinumab, anti-p19, or anakinra 6

Supplemental information: mIBD REPORT standards worksheet example checklist 9

Supplemental References 11

Supplementary Table 1. Summary of included articles by gene defect

Gene	PMID
DOCK2	36836791[1], 36947335[2], 29204803[3], 30826364[4], 26083206[5], 30838481[6], 33928462[7]
CDC42	31601675[8], 31271789[9]
WAS	27113846[10], 12728121[11], 32463623[12], 29358862[13], 36214981[14], 18546019[15], 24622513[16], 35075289[17]
WIPF1	27742395[18], 28903942[19]
ARPC1B	30771411[20]
PIK3CD	31073077[21], 37202577[22]
SYK	33782605[23]
PIK3R1	37202577[22], 22351933[24], 25488983[25]
BTK	34029777[26], 33259974[27], 28236219[28], 28261296[29]
CYBC1	30361506[30]
PRKCD	NA
CYBA	21058909[31], 15940051[32], 27537055[33]
NCF4	31027832[34]
NCF1	21058909[31], 28919373[35]
CYBB	26464403[36], 27465505[37], 33338684[38], 21058909[31], 26052777[39], 22015491[40], 12634733[41], 30094936[42], 29018441[43], 31988463[44]
NCF2	26052777[39]
SLC37A4	35433171[45], 32294159[46], 34215305[47], 35822097[48], 36039216[49], 36507137[50], 34307579[51], 18172743[52], 11383585[53], 25881301[54], 18206704[55]
G6PC3	32930428[56], 31157858[57], 34305938[58]
LACC1	NA
FMNL2	NA
NPC1	35694196[59], 31352388[60], 35741735[61], 37168644[62]
TRIM22	26836588[63]
XIAP	27815752[64], 22228567[65], 23973892[66], 23131490[67], 25943627[68], 25412586[69], 21173700[70], 24572142[71], 29531467[72], 26671016[73], 24942029[74], 31232887[75], 32540394[76], 26581487[77], 33460440[78], 29312354[79], 32514016[80], 34920138[81], 29894681[82], 37006985[83], 25023582[84], 35124956[85], 26584814[86], 35304666[87]
CARD8	29408806[88]
IL10	22549091[89], 29023267[90], 20934598[91]
IL10RA	24216686[92], 22549091[89], 23158016[93], 24001973[94], 24519095[95], 28597964[96], 35187668[97]
IL10RB	24216686[92], 22549091[89], 23158016[93], 29531467[72], 19890111[98], 25373860[99], 27336593[100], 35366317[101], 27699073[102], 35187668[97], 35246832[103]
ITGB2	37006985[83], 8944573[104]
ADA2	25457153[105], 28974505[106], 31856934[107], 31945408[108], 33705245[109], 34057601[110]
STXBP3	33891011[111]
CD3G	18482219[112]
IL2RG	32490820[113]
RAG2	NA

RAG1	NA
ZAP70	36669607[114], 28124082[115], 26783323[116]
DCLRE1C	19967552[117]
ADA	NA
LIG4	NA
STAT1	NA
STAT3	28153428[118]
PTPN2	32721438[119]
IL2RA	NA
IL2RB	31040185[120]
MALT1	27253662[121]
FOXP3	32084423[122], 27302973[123], 29312905[124], 35186001[125], 30385752[126], 23060872[127], 18481161[128], 17115064[129]
CTLA4	25367873[130], 33965572[131], 29729943[132], 37205940[133], 27102614[134], 25213377[135], 34111452[136], 27908448[137]
LRBA	32084423[122], 27302973[123], 25479458[138], 28956255[139], 26206937[140], 36214981[14], 32915433[141], 35748740[142], 36569874[143], 35246832[103], 28884992[144], 28551707[145], 28197149[146], 29140941[147], 34368306[148], 26745254[149], 37006985[83], 31887391[150]
BACH2	NA
IL21	NA
CD40LG	24845792[151], 29303623[152]
CARMIL2	32201938[153], 34287962[154], 33723309[155], 31115454[156]
MASP2	NA
ANKZF1	36857589[157]
SAMD9	NA
ICOS	19380800[158]
IKZF1	NA
IL37	NA
SCGN	31663849[159]
ELF4	36823308[160]
TTC7A	35783276[161], 35627206[162], 28936210[163], 31616743[164], 29879038[165], 27418642[166], 25546680[167], 23830146[168]
PI4KA	NA
WNT2B	29909964[169]
AGR2	NA
SLC9A3	NA
SLC26A3	33770165[170]
GUCY2C	NA
TTC37	25194001[171], 28027214[172], 26945392[173]
SKIV2L	25194001[171], 37168478[174]
COL7A1	NA
FERMT1	24002169[175]
ALPI	29567797[176]
RIPK1	36466854[177], 30591564[178], 32181283[179]
CASP8	30267714[180], 37038193[181]
RBCK1	23104095[182]
TNFAIP3	34427832[183], 29241730[184], 29317407[185], 34030699[186], 32719680[187],

	32514655[188], 31767699[189], 36641803[190], 36467491[191], 34808442[192], 33549127[193], 33446651[194], 31299923[195], 31164164[196], 32011208[197]
STIM1	NA
BCL10	NA
CARD11	36443613[198], 33872653[199]
RELA	28600438[200], 32487952[201]
NFKBIA	23708964[202], 14523047[203]
IKBKG	28513998[204], 21993693[205], 31713830[206], 28679735[207]
HPS1	30634918[208], 24989352[209], 30833808[210], 20543722[211]
HPS3	NA
HPS4	29600982[212]
RTEL1	27128385[213]
DKC1	NA
NOP10	NA
TGFB1	29483653[214]
TGFBR1	27508510[215], 24486179[216]
TGFBR2	27508510[215], 24486179[216], 37168749[217]
NLRC4	27876626[218], 34783940[219]
POLA1	NA
TYMP	NA
ITCH	20170897[220], 36338154[221], 33894394[222]
MVK	34525209[223], 21708801[224], 30862258[225], 23979089[226]
CD55	28657829[227], 33398182[228], 28657861[229], 30418410[230], 35314883[231], 33215321[232]
SIRT1	NA
HPSA1L	28126021[233]
SLCO2A1	35611666[234], 36480694[235], 31878983[236]
TRNT1	29358286[237], 27531075[238], 36937953[239]
NFAT5	NA
IRF2BP2	37350971[240], 30941118[241]

Supplementary Figure 1. Quantified therapeutic response ranges for therapeutics used in mIBD.

Percentage of mIBD patients with complete and incomplete therapeutic responses, implementing 95% confidence intervals.

Infliximab, adalimumab, IL1 antagonist (anakinra, canakinumab) across multiple mIBD conditions. WAS-like (WAS, WIPF1, ARPC1B), CGD (CYBC1, CYBA, CYBB, NCF1, NCF2, NCF4, PRKCD), congenital neutropenia (SLC37A4, G6PC3), IL10 signalling defects (IL10, IL10RA, IL10RB), IPEX-like disorders, congenital diarrhea (SLC9A3, SLC26A3, GUCY2C), HPS (HPS1, HPS4), LDS (TGFBR1, TGFBR2)

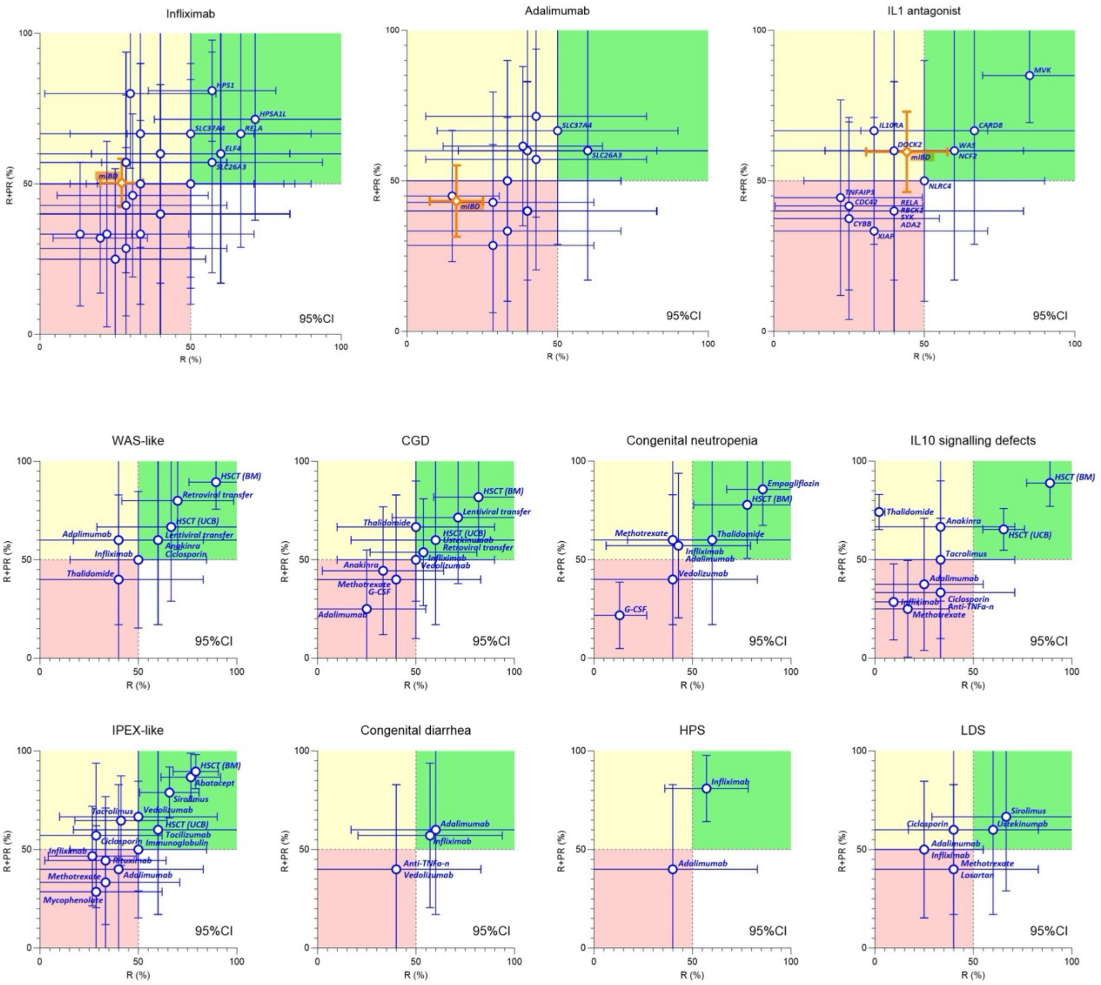

Supplementary case reports: Case history of patients with IL10 defects treated with ustekinumab or anti-p19 therapeutics

Case 1 (from Dr. Batya)

This is a 14-year-old boy with infantile-onset monogenic inflammatory bowel disease (mIBD), with symptoms that began around one month of age. Diagnostic endoscopy at three years old revealed features consistent with Crohn's disease (CD) (L2B3p), and genetic testing confirmed an IL10RA mutation. He did not respond well to thiopurine or infliximab, and at 7.5 years old, he was started on ustekinumab, which he received for four months until his last follow-up. While his clinical symptoms (abdominal pain, rectal bleeding, stool frequency, and consistency) began to improve, his laboratory data showed no significant improvement. Overall, his disease exhibited a "partial response" to ustekinumab.

Case 2 (from Dr. Lau)

This 16-year-old boy began exhibiting mIBD-related symptoms, including fever, oral ulcerations, blood-stained diarrhoea, perianal ulceration, and growth retardation, at just two weeks of age. At around three years old, genetic testing revealed compound heterozygous missense mutations in the extracellular domain of IL-10R1 (c.251C>T, p.T84I; c.301C>T, p.R101W). By that time, he had already been treated for Crohn's disease with a combination of therapies, including corticosteroids, azathioprine, methotrexate, and infliximab. Over the years, his therapeutic regimen was adjusted (including adalimumab, risankizumab, upadacitinib, ruxolitinib, tocilizumab, thalidomide, and intravenous immunoglobulin), and his colitis activity was better controlled until 2023, when a major relapse occurred, potentially due to the prior cessation of JAK inhibitors and additional food triggers. This flare was managed by reintroducing JAK inhibitors, pulse corticosteroid therapy, total parenteral nutrition, and trying ustekinumab. He responded well to ustekinumab and gradually improved towards remission. He has achieved a good "partial response" with his combination therapies: abdominal pain has subsided, stool patterns have improved, and the perianal lesions are healing. Currently, he has received a 10/10 mismatched unrelated donor peripheral blood stem cell transplant in Shanghai and has remained well and engrafted after around six months of follow-up.

Case 3 (from Oxford/Qatar)

This 38-year-old female was born into a consanguineous family with multiple members affected by fatal colitis. She presented with rectal bleeding and intractable perianal disease from 3 months of age, requiring subtotal colectomy, ileostomy, and the creation of a Hartmann pouch at 9 months. Diagnosed with Crohn's disease (CD, A1L3B3p), characterized by enterocutaneous fistulation and perineal cutaneous disease, she experienced multiple

ineffective treatments, including sulfasalazine, corticosteroids, azathioprine, methotrexate, certolizumab, infliximab (partial response; later complicated by Hodgkin's lymphoma), adalimumab (partial response), and ustekinumab (suspected primary non-response). Genetic testing revealed a homozygous splice-site mutation in IL10RB (IVS311G>C), leading to a frameshift and a predicted truncated IL-10R2 protein (Leu59fsX72). Due to the development of high anti-drug antibody levels, infliximab was discontinued and replaced with subcutaneous risankizumab in 2023, which has since achieved acceptable clinical, endoscopic, and radiological responses.

Case 4 (from Oxford)

This 30-year-old female was born to a consanguineous family (parents were first cousins; South Asian ethnicity). She had diarrhea from birth and gradually developed perianal disease. At three years of age, she was diagnosed with CD and underwent ileostomy formation. The stoma was reversed at age five but re-created with a subtotal colectomy at age eight. Over the following years, she required multiple additional surgeries, including exploratory laparotomy with terminal ileum resection for gastrointestinal obstruction, and completion proctectomy at age 23. Her disease course was complicated by folliculitis, flexural eczema, erythema nodosum, renal failure, and an episode of colonic perforation requiring ICU admission. She failed to respond to 6-mercaptopurine, adalimumab (discontinued due to injection-site intolerance), and infliximab (partial response without improvement in fistulizing and perianal disease). At age 24, genetic and functional studies confirmed an IL10RB mutation; however, she declined to have allogeneic HSCT. Anakinra was initiated in January 2021 but was discontinued three months later due to severe injection-site reactions. Currently, she has declined IBD-directed therapy but intermittently receives courses of advanced antibiotics for her perineal complications, which include vulvar lymphedema, labial hypertrophy, fissures, ulcers, and abscesses with discharge.

Case 5 (from Dr. Quynh)

This 16-month-old boy was born full-term to a non-consanguineous Vietnamese family, with no significant perinatal complications. At 2 months of age, he developed vomiting and diarrhea, accompanied by abdominal pain, intermittent prolonged fever without an identifiable infectious focus, anemia, leukocytosis, and elevated inflammatory markers (CRP, IL-1β, IL-18, IL-23). Endoscopic evaluation demonstrated colitis with no upper gastrointestinal involvement. Genetic testing identified a homozygous IL10RA mutation (c.421G>A, p.(Gly141Arg)), with each parent carrying one variant allele. He received multiple therapies without success, including modified enteral feeding (hydrolyzed or dairy-free formulas), corticosteroids, mesalazine, azathioprine, and infliximab (administered for 8 months without improvement). A 1.5-month trial of anakinra likewise failed to improve either clinical

symptoms or laboratory markers. Subsequent treatment with IVIG and tocilizumab led to the resolution of fever and better tolerance of enteral feeding, and he is currently awaiting an unrelated umbilical cord blood stem cell transplant.

Supplemental information: mIBD REPORT standards worksheet example checklist

mIBD REPORT (**m**onogenic **I**nflammatory **B**owel **D**isease **R**eport **E**xtended **P**henotype and **O**utcome of t**R**ea**T**ments)

Item			Description/Example
**Step 1: Patient identification**			
1.1	☐	Patient identification code	Gene-Identifier number (e.g., NPC1-35694196)
1.2	☐	Gender	M(male)/F(female)
1.3	☐	Age (current)	Yr/mo
**Step 2: Molecular diagnostics**			
2.1	☐	Gene name	e.g., NPC1
2.2	☐	Causative variant (protein, DNA, 2nd variant)	e.g., p.L1244P, c.C562T
**Step 3. Clinical characteristics of IBD**			
3.1	☐	Clinical phenotype	CD/UC/IBDU/Others
3.2	☐	Disease location/extent/behavior/perianal disease	Paris/Montreal: L1-L4, E1-E3, B1-B3, p
3.3	☐	Histology confirmation at diagnosis	Y/N
3.4	☐	Age of first suspicion of IBD	Yr/mo
3.5	☐	Age of the diagnostic (index) endoscopy	Yr/mo
3.6	☐	History of IBD related surgery	Bowel resection, pouch/ostomy creation, etc.
3.7	☐	Extraintestinal manifestations (EIM)<br>☐ Classical EIM<br>☐ Syndromic manifestation<br>☐ Please specify: _________________	Classical (arthritis, bone, eyes, skin, liver, etc.); syndromic (infection susceptibility etc.)
**Step 4: Medication history**			
4.1	☐	Indicate Y/N for all listed medications	P (past use)/C (current use)/I (Intolerance)
**Step 5: Major medication and the IBD-related response**			
5.1	☐	Drug name, dose, route	e.g., 5mg/kg Q8W, IV
5.2	☐	Therapy setting	P (per protocol)/O (off license)/<br>T (prospective trial)/C (compassionate use)
5.3	☐	Date and age (start and follow-up)	Baseline, week 10 (8-12), 26 (20-32), 52 (40-65)
5.4	☐	Anthropometric/growth assessment	Height (cm)/Weight (kg) on all timepoints
5.5	☐	IBD-related response (see 5.6-5.14)	
5.6	☐	Global response	R (remission)/PR (partial remission)/A (active)
5.7	☐	Severity scores	Child (wPCDAI/PUCAI), adult (PRO2/CDAI)
5.8	☐	Biomarker response	WBC, CRP, fecal calprotectin
5.9	☐	Endoscopy response *(optional)**	Endoscopic Mayo score, SES-CD
5.10	☐	Histology healing *(optional)**	Nancy index
5.11	☐	Quality of life assessment *(optional)*	IBDQ or other validated scores
5.12	☐	Other pathology or disease-specific response	Y/N, with description, examples
5.13	☐	Side effect and adverse event	Y/N, with description

5.14	☐	EIM response	Y/N, with description (resolution or adverse event)
**Step 6: Current disease activity**			
6.1	☐	Indicate patient's latest overall IBD disease activity	Deep remission (DR)<br>Clinical remission (CR)<br>Partial remission (PR)<br>Active disease (A)

* Suggested criteria for scoring:

1. Endoscopic Mayo score (eMayo): response (eMayo grade 0, or a reduction of ≥ 2 grades); partial response (eMayo grade 1, or a reduction of 1 grade).
2. Simple Endoscopic Score for Crohn's Disease (SES-CD): response (SES-CD ≤ 2, or a reduction of ≥ 50% from baseline); partial response (SES-CD reduction of ≥ 25% from baseline).
3. Nancy index (NI): response (NI ≤ 1); partial response (NI reduction but not yet ≤ 1).

Supplemental References

1. Reiff DD, Zhang M, Cron RQ. DOCK2 Mutation and Recurrent Hemophagocytic Lymphohistiocytosis. *Life (Basel)*. Feb 3 2023;13(2)doi:10.3390/life13020434
2. Noble AJ, Nowak JK, Adams AT, Uhlig HH, Satsangi J. Defining Interactions Between the Genome, Epigenome, and the Environment in Inflammatory Bowel Disease: Progress and Prospects. *Gastroenterology*. Jul 2023;165(1):44–60.e2. doi:10.1053/j.gastro.2023.03.238
3. Alizadeh Z, Mazinani M, Shakerian L, Nabavi M, Fazlollahi MR. DOCK2 Deficiency in a Patient with Hyper IgM Phenotype. *J Clin Immunol*. Jan 2018;38(1):10–12. doi:10.1007/s10875-017-0468-5
4. Alosaimi MF, Shendi H, Beano A, et al. T-cell mitochondrial dysfunction and lymphopenia in DOCK2-deficient patients. *J Allergy Clin Immunol*. Jul 2019;144(1):306–309.e2. doi:10.1016/j.jaci.2019.02.020
5. Dobbs K, Domínguez Conde C, Zhang SY, et al. Inherited DOCK2 Deficiency in Patients with Early-Onset Invasive Infections. *N Engl J Med*. Jun 18 2015;372(25):2409–22. doi:10.1056/NEJMoa1413462
6. Moens L, Gouwy M, Bosch B, et al. Human DOCK2 Deficiency: Report of a Novel Mutation and Evidence for Neutrophil Dysfunction. *J Clin Immunol*. Apr 2019;39(3):298–308. doi:10.1007/s10875-019-00603-w
7. D'Astous-Gauthier K, Desjardins A, Marois L, Falcone EL, Chapdelaine H. DOCK2 Deficiency Diagnosed 18 Years After Hematopoietic Stem Cell Transplantation. *J Clin Immunol*. Aug 2021;41(6):1400–1402. doi:10.1007/s10875-021-01040-4
8. Lam MT, Coppola S, Krumbach OHF, et al. A novel disorder involving dyshematopoiesis, inflammation, and HLH due to aberrant CDC42 function. *J Exp Med*. Dec 2 2019;216(12):2778–2799. doi:10.1084/jem.20190147
9. Gernez Y, de Jesus AA, Alsaleem H, et al. Severe autoinflammation in 4 patients with C-terminal variants in cell division control protein 42 homolog (CDC42) successfully treated with IL-1β inhibition. *J Allergy Clin Immunol*. Oct 2019;144(4):1122–1125.e6. doi:10.1016/j.jaci.2019.06.017
10. Brigida I, Scaramuzza S, Lazarevic D, et al. A novel genomic inversion in Wiskott-Aldrich-associated autoinflammation. *J Allergy Clin Immunol*. Aug 2016;138(2):619–622.e7. doi:10.1016/j.jaci.2016.03.007
11. Dupuis-Girod S, Medioni J, Haddad E, et al. Autoimmunity in Wiskott-Aldrich syndrome: risk factors, clinical features, and outcome in a single-center cohort of 55 patients. *Pediatrics*. May 2003;111(5 Pt 1):e622–7. doi:10.1542/peds.111.5.e622
12. Ashton JJ, Mossotto E, Stafford IS, et al. Genetic Sequencing of Pediatric Patients Identifies Mutations in Monogenic Inflammatory Bowel Disease Genes that Translate to Distinct Clinical Phenotypes. *Clin Transl Gastroenterol*. Feb 2020;11(2):e00129.

doi:10.14309/ctg.0000000000000129
13. Ohya T, Yanagimachi M, Iwasawa K, et al. Childhood-onset inflammatory bowel diseases associated with mutation of Wiskott-Aldrich syndrome protein gene. *World J Gastroenterol*. Dec 28 2017;23(48):8544–8552. doi:10.3748/wjg.v23.i48.8544
14. Moser LM, Fekadu J, Willasch A, et al. Treatment of inborn errors of immunity patients with inflammatory bowel disease phenotype by allogeneic stem cell transplantation. *Br J Haematol*. Mar 2023;200(5):595–607. doi:10.1111/bjh.18497
15. Cannioto Z, Berti I, Martelossi S, et al. IBD and IBD mimicking enterocolitis in children younger than 2 years of age. *Eur J Pediatr*. Feb 2009;168(2):149–55. doi:10.1007/s00431-008-0721-2
16. Braun CJ, Boztug K, Paruzynski A, et al. Gene therapy for Wiskott-Aldrich syndrome--long-term efficacy and genotoxicity. *Sci Transl Med*. Mar 12 2014;6(227):227ra33. doi:10.1126/scitranslmed.3007280
17. Magnani A, Semeraro M, Adam F, et al. Long-term safety and efficacy of lentiviral hematopoietic stem/progenitor cell gene therapy for Wiskott-Aldrich syndrome. *Nat Med*. Jan 2022;28(1):71–80. doi:10.1038/s41591-021-01641-x
18. Al-Mousa H, Hawwari A, Al-Ghonaium A, et al. Hematopoietic stem cell transplantation corrects WIP deficiency. *J Allergy Clin Immunol*. Mar 2017;139(3):1039–1040.e4. doi:10.1016/j.jaci.2016.08.036
19. Pfajfer L, Seidel MG, Houmadi R, et al. WIP deficiency severely affects human lymphocyte architecture during migration and synapse assembly. *Blood*. Oct 26 2017;130(17):1949–1953. doi:10.1182/blood-2017-04-777383
20. Volpi S, Cicalese MP, Tuijnenburg P, et al. A combined immunodeficiency with severe infections, inflammation, and allergy caused by ARPC1B deficiency. *J Allergy Clin Immunol*. Jun 2019;143(6):2296–2299. doi:10.1016/j.jaci.2019.02.003
21. Swan DJ, Aschenbrenner D, Lamb CA, et al. Immunodeficiency, autoimmune thrombocytopenia and enterocolitis caused by autosomal recessive deficiency of PIK3CD-encoded phosphoinositide 3-kinase δ. *Haematologica*. Oct 2019;104(10):e483–e486. doi:10.3324/haematol.2018.208397
22. Lee WI, Chen CC, Chen SH, et al. Clinical Features and Genetic Analysis of Taiwanese Primary Immunodeficiency Patients with Prolonged Diarrhea and Monogenetic Inflammatory Bowel Disease. *J Clin Immunol*. Aug 2023;43(6):1455–1467. doi:10.1007/s10875-023-01503-w
23. Wang L, Aschenbrenner D, Zeng Z, et al. Gain-of-function variants in SYK cause immune dysregulation and systemic inflammation in humans and mice. *Nat Genet*. Apr 2021;53(4):500–510. doi:10.1038/s41588-021-00803-4
24. Conley ME, Dobbs AK, Quintana AM, et al. Agammaglobulinemia and absent B lineage cells in a patient lacking the p85α subunit of PI3K. *J Exp Med*. Mar 12 2012;209(3):463–70.

doi:10.1084/jem.20112533
25. Lucas CL, Zhang Y, Venida A, et al. Heterozygous splice mutation in PIK3R1 causes human immunodeficiency with lymphoproliferation due to dominant activation of PI3K. *J Exp Med*. Dec 15 2014;211(13):2537–47. doi:10.1084/jem.20141759
26. Khan F, Person H, Dekio F, et al. Crohn's-like Enteritis in X-Linked Agammaglobulinemia: A Case Series and Systematic Review. *J Allergy Clin Immunol Pract*. Sep 2021;9(9):3466–3478. doi:10.1016/j.jaip.2021.04.070
27. King JR, Grover Z, Irani N, McLean-Tooke A. Vedolizumab is safe and effective in the treatment of X-linked agammaglobulinemia-associated inflammatory bowel disease. *J Allergy Clin Immunol Pract*. Feb 2021;9(2):1006–1007. doi:10.1016/j.jaip.2020.11.040
28. Barmettler S, Otani IM, Minhas J, et al. Gastrointestinal Manifestations in X-linked Agammaglobulinemia. *J Clin Immunol*. Apr 2017;37(3):287–294. doi:10.1007/s10875-017-0374-x
29. Pac M, Bernatowska EA, Kierkuś J, et al. Gastrointestinal disorders next to respiratory infections as leading symptoms of X-linked agammaglobulinemia in children - 34-year experience of a single center. *Arch Med Sci*. Mar 1 2017;13(2):412–417. doi:10.5114/aoms.2016.60338
30. Arnadottir GA, Norddahl GL, Gudmundsdottir S, et al. A homozygous loss-of-function mutation leading to CYBC1 deficiency causes chronic granulomatous disease. *Nat Commun*. Oct 25 2018;9(1):4447. doi:10.1038/s41467-018-06964-x
31. Uzel G, Orange JS, Poliak N, Marciano BE, Heller T, Holland SM. Complications of tumor necrosis factor-α blockade in chronic granulomatous disease-related colitis. *Clin Infect Dis*. Dec 15 2010;51(12):1429–34. doi:10.1086/657308
32. Güngör T, Halter J, Klink A, et al. Successful low toxicity hematopoietic stem cell transplantation for high-risk adult chronic granulomatous disease patients. *Transplantation*. Jun 15 2005;79(11):1596–606. doi:10.1097/01.tp.0000163466.73485.5e
33. Ashton JJ, Andreoletti G, Coelho T, et al. Identification of Variants in Genes Associated with Single-gene Inflammatory Bowel Disease by Whole-exome Sequencing. *Inflamm Bowel Dis*. Oct 2016;22(10):2317–27. doi:10.1097/mib.0000000000000890
34. Wright M, Chandrakasan S, Okou DT, et al. Early Onset Granulomatous Colitis Associated with a Mutation in NCF4 Resolved with Hematopoietic Stem Cell Transplantation. *J Pediatr*. Jul 2019;210:220–225. doi:10.1016/j.jpeds.2019.03.042
35. Campbell N, Chapdelaine H. Treatment of chronic granulomatous disease-associated fistulising colitis with vedolizumab. *J Allergy Clin Immunol Pract*. Nov–Dec 2017;5(6):1748–1749. doi:10.1016/j.jaip.2017.07.035
36. Hauck F, Koletzko S, Walz C, et al. Diagnostic and Treatment Options for Severe IBD in Female X-CGD Carriers with Non-random X-inactivation. *J Crohns Colitis*. Jan 2016;10(1):112–5. doi:10.1093/ecco-jcc/jjv186

37. Butte MJ, Park KT, Lewis DB. Treatment of CGD-associated Colitis with the IL-23 Blocker Ustekinumab. *J Clin Immunol*. Oct 2016;36(7):619–20. doi:10.1007/s10875-016-0318-x
38. Falcone EL, Han Y, Kreuzburg S, et al. Exclusive enteral nutrition induced sustained changes in the microbiota and improved inflammatory bowel disease in a pediatric patient with chronic granulomatous disease. *J Allergy Clin Immunol Pract*. Feb 2021;9(2):1011–1014.e2. doi:10.1016/j.jaip.2020.12.014
39. Hahn KJ, Ho N, Yockey L, et al. Treatment With Anakinra, a Recombinant IL-1 Receptor Antagonist, Unlikely to Induce Lasting Remission in Patients With CGD Colitis. *Am J Gastroenterol*. Jun 2015;110(6):938–9. doi:10.1038/ajg.2015.135
40. Kato K, Kojima Y, Kobayashi C, et al. Successful allogeneic hematopoietic stem cell transplantation for chronic granulomatous disease with inflammatory complications and severe infection. *Int J Hematol*. Nov 2011;94(5):479–82. doi:10.1007/s12185-011-0932-6
41. Bhattacharya A, Slatter M, Curtis A, et al. Successful umbilical cord blood stem cell transplantation for chronic granulomatous disease. *Bone Marrow Transplant*. Mar 2003;31(5):403–5. doi:10.1038/sj.bmt.1703863
42. Zurro NB, Tavares de Albuquerque JA, França TT, et al. A novel mutation in CYBB gene in a patient with chronic colitis and recurrent pneumonia due to X-linked chronic granulomatous disease. *Pediatr Blood Cancer*. Dec 2018;65(12):e27382. doi:10.1002/pbc.27382
43. Labrosse R, Abou-Diab J, Blincoe A, et al. Very Early-Onset Inflammatory Manifestations of X-Linked Chronic Granulomatous Disease. *Front Immunol*. 2017;8:1167. doi:10.3389/fimmu.2017.01167
44. Kohn DB, Booth C, Kang EM, et al. Lentiviral gene therapy for X-linked chronic granulomatous disease. *Nat Med*. Feb 2020;26(2):200–206. doi:10.1038/s41591-019-0735-5
45. Kaczor M, Greczan M, Kierus K, et al. Sodium-glucose cotransporter type 2 channel inhibitor: Breakthrough in the treatment of neutropenia in patients with glycogen storage disease type 1b? *JIMD Rep*. May 2022;63(3):199–206. doi:10.1002/jmd2.12278
46. Wortmann SB, Van Hove JLK, Derks TGJ, et al. Treating neutropenia and neutrophil dysfunction in glycogen storage disease type Ib with an SGLT2 inhibitor. *Blood*. Aug 27 2020;136(9):1033–1043. doi:10.1182/blood.2019004465
47. Rossi A, Miele E, Fecarotta S, et al. Crohn disease-like enterocolitis remission after empagliflozin treatment in a child with glycogen storage disease type Ib: a case report. *Ital J Pediatr*. Jul 2 2021;47(1):149. doi:10.1186/s13052-021-01100-w
48. Tallis E, Karsenty CL, Grimes AB, et al. Untargeted metabolomic profiling in a patient with glycogen storage disease Ib receiving empagliflozin treatment. *JIMD Rep*. Jul 2022;63(4):309–315. doi:10.1002/jmd2.12304
49. Makrilakis K, Barmpagianni A, Veiga-da-Cunha M. Repurposing of Empagliflozin as a Possible Treatment for Neutropenia and Inflammatory Bowel Disease in Glycogen Storage

Disease Type Ib: A Case Report. *Cureus*. Jul 2022;14(7):e27264. doi:10.7759/cureus.27264
50. Hexner-Erlichman Z, Veiga-da-Cunha M, Zehavi Y, et al. Favorable outcome of empagliflozin treatment in two pediatric glycogen storage disease type 1b patients. *Front Pediatr*. 2022;10:1071464. doi:10.3389/fped.2022.1071464
51. Gong YZ, Zhong XM, Zou JZ. Infliximab treatment of glycogenosis Ib with Crohn's-like enterocolitis: A case report. *World J Clin Cases*. Jul 6 2021;9(19):5280–5286. doi:10.12998/wjcc.v9.i19.5280
52. Davis MK, Rufo PA, Polyak SF, Weinstein DA. Adalimumab for the treatment of Crohn-like colitis and enteritis in glycogen storage disease type Ib. *J Inherit Metab Dis*. Dec 2008;31 Suppl 3:505–9. doi:10.1007/s10545-007-0774-9
53. Yamaguchi T, Ihara K, Matsumoto T, et al. Inflammatory bowel disease-like colitis in glycogen storage disease type 1b. *Inflamm Bowel Dis*. May 2001;7(2):128–32. doi:10.1097/00054725-200105000-00008
54. Volz MS, Nassir M, Treese C, et al. Inflammatory bowel disease (IBD)-like disease in a case of a 33-year old man with glycogenosis 1b. *BMC Gastroenterol*. Apr 8 2015;15:45. doi:10.1186/s12876-015-0271-9
55. Pierre G, Chakupurakal G, McKiernan P, Hendriksz C, Lawson S, Chakrapani A. Bone marrow transplantation in glycogen storage disease type 1b. *J Pediatr*. Feb 2008;152(2):286–8. doi:10.1016/j.jpeds.2007.09.031
56. Goenka A, Doherty JA, Al-Farsi T, et al. Neutrophil dysfunction triggers inflammatory bowel disease in G6PC3 deficiency. *J Leukoc Biol*. Jun 2021;109(6):1147–1154. doi:10.1002/jlb.5ab1219-699rr
57. Bolton C, Burch N, Morgan J, et al. Remission of Inflammatory Bowel Disease in Glucose-6-Phosphatase 3 Deficiency by Allogeneic Haematopoietic Stem Cell Transplantation. *J Crohns Colitis*. Jan 1 2020;14(1):142–147. doi:10.1093/ecco-jcc/jjz112
58. Dai R, Lv G, Li W, et al. Altered Functions of Neutrophils in Two Chinese Patients With Severe Congenital Neutropenia Type 4 Caused by G6PC3 Mutations. *Front Immunol*. 2021;12:699743. doi:10.3389/fimmu.2021.699743
59. Williams I, Pandey S, Haller W, et al. Anti-TNF therapy for inflammatory bowel disease in patients with neurodegenerative Niemann-Pick disease Type C. *Wellcome Open Res*. 2022;7:11. doi:10.12688/wellcomeopenres.16986.1
60. Dike CR, Bernat J, Bishop W, DeGeeter C. Niemann-Pick disease type C presenting as very early onset inflammatory bowel disease. *BMJ Case Rep*. Jul 27 2019;12(7)doi:10.1136/bcr-2019-229780
61. Azab B, Rabab'h O, Aburizeg D, et al. Potential Composite Digenic Contribution of NPC1 and NOD2 Leading to Atypical Lethal Niemann-Pick Type C with Initial Crohn's Disease-like Presentation: Genotype-Phenotype Correlation Study. *Genes (Basel)*. May 29 2022;13(6)doi:10.3390/genes13060973

62. Hudson AS, Almeida P, Huynh HQ. Dual Biologic Therapy in a Patient With Niemann-Pick Type C and Crohn Disease: A Case Report and Literature Review. *JPGN Rep*. Aug 2022;3(3):e225. doi:10.1097/pg9.0000000000000225
63. Li Q, Lee CH, Peters LA, et al. Variants in TRIM22 That Affect NOD2 Signaling Are Associated With Very-Early-Onset Inflammatory Bowel Disease. *Gastroenterology*. May 2016;150(5):1196–1207. doi:10.1053/j.gastro.2016.01.031
64. Ono S, Okano T, Hoshino A, et al. Hematopoietic Stem Cell Transplantation for XIAP Deficiency in Japan. *J Clin Immunol*. Jan 2017;37(1):85–91. doi:10.1007/s10875-016-0348-4
65. Yang X, Kanegane H, Nishida N, et al. Clinical and genetic characteristics of XIAP deficiency in Japan. *J Clin Immunol*. Jun 2012;32(3):411–20. doi:10.1007/s10875-011-9638-z
66. Speckmann C, Lehmberg K, Albert MH, et al. X-linked inhibitor of apoptosis (XIAP) deficiency: the spectrum of presenting manifestations beyond hemophagocytic lymphohistiocytosis. *Clin Immunol*. Oct 2013;149(1):133–41. doi:10.1016/j.clim.2013.07.004
67. Marsh RA, Rao K, Satwani P, et al. Allogeneic hematopoietic cell transplantation for XIAP deficiency: an international survey reveals poor outcomes. *Blood*. Feb 7 2013;121(6):877–83. doi:10.1182/blood-2012-06-432500
68. Dziadzio M, Ammann S, Canning C, et al. Symptomatic males and female carriers in a large Caucasian kindred with XIAP deficiency. *J Clin Immunol*. Jul 2015;35(5):439–44. doi:10.1007/s10875-015-0166-0
69. Tsuma Y, Imamura T, Ichise E, et al. Successful treatment of idiopathic colitis related to XIAP deficiency with allo-HSCT using reduced-intensity conditioning. *Pediatr Transplant*. Feb 2015;19(1):E25–8. doi:10.1111/petr.12405
70. Worthey EA, Mayer AN, Syverson GD, et al. Making a definitive diagnosis: successful clinical application of whole exome sequencing in a child with intractable inflammatory bowel disease. *Genet Med*. Mar 2011;13(3):255–62. doi:10.1097/GIM.0b013e3182088158
71. Zeissig Y, Petersen BS, Milutinovic S, et al. XIAP variants in male Crohn's disease. *Gut*. Jan 2015;64(1):66–76. doi:10.1136/gutjnl-2013-306520
72. Fang YH, Luo YY, Yu JD, Lou JG, Chen J. Phenotypic and genotypic characterization of inflammatory bowel disease in children under six years of age in China. *World J Gastroenterol*. Mar 7 2018;24(9):1035–1045. doi:10.3748/wjg.v24.i9.1035
73. Girardelli M, Arrigo S, Barabino A, et al. The diagnostic challenge of very early-onset enterocolitis in an infant with XIAP deficiency. *BMC Pediatr*. Dec 15 2015;15:208. doi:10.1186/s12887-015-0522-5
74. Varghese AS, Lee H, Bonney D, Hughes S, Wynn R. Complications of Reduced Intensity Conditioning HSCT for XIAP Deficiency (Alloimmune Cytopenias and HLH) Successfully Managed With Donor Lymphocyte Infusion. *J Pediatr Hematol Oncol*. Apr 2015;37(3):e198–9. doi:10.1097/mph.0000000000000191
75. Lekbua A, Ouahed J, O'Connell AE, et al. Risk-factors Associated With Poor Outcomes in

VEO-IBD Secondary to XIAP Deficiency: A Case Report and Literature Review. *J Pediatr Gastroenterol Nutr*. Jul 2019;69(1):e13–e18. doi:10.1097/mpg.0000000000002297
76. Takeuchi I, Kawai T, Nambu M, et al. X-linked inhibitor of apoptosis protein deficiency complicated with Crohn's disease-like enterocolitis and Takayasu arteritis: A case report. *Clin Immunol*. Aug 2020;217:108495. doi:10.1016/j.clim.2020.108495
77. Kelsen JR, Dawany N, Martinez A, et al. A de novo whole gene deletion of XIAP detected by exome sequencing analysis in very early onset inflammatory bowel disease: a case report. *BMC Gastroenterol*. Nov 18 2015;15:160. doi:10.1186/s12876-015-0394-z
78. Chang I, Park S, Lee HJ, et al. Interpretation of XIAP Variants of Uncertain Significance in Paediatric Patients with Refractory Crohn's Disease. *J Crohns Colitis*. Aug 2 2021;15(8):1291–1304. doi:10.1093/ecco-jcc/jjab013
79. Cifaldi C, Chiriaco M, Di Matteo G, et al. Novel X-Linked Inhibitor of Apoptosis Mutation in Very Early-Onset Inflammatory Bowel Disease Child Successfully Treated with HLA-Haploidentical Hemapoietic Stem Cells Transplant after Removal of αβ(+) T and B Cells. *Front Immunol*. 2017;8:1893. doi:10.3389/fimmu.2017.01893
80. Parackova Z, Milota T, Vrabcova P, et al. Novel XIAP mutation causing enhanced spontaneous apoptosis and disturbed NOD2 signalling in a patient with atypical adult-onset Crohn's disease. *Cell Death Dis*. Jun 8 2020;11(6):430. doi:10.1038/s41419-020-2652-4
81. He C, Li H, Zhou P, Zhang W, Li J, Li M. A novel XIAP mutation in an adult Chinese patient with refractory Crohn's disease. *Clin Res Hepatol Gastroenterol*. Apr 2022;46(4):101845. doi:10.1016/j.clinre.2021.101845
82. Quaranta M, Wilson R, Gonçalves Serra E, et al. Consequences of Identifying XIAP Deficiency in an Adult Patient With Inflammatory Bowel Disease. *Gastroenterology*. Jul 2018;155(1):231–234. doi:10.1053/j.gastro.2018.03.069
83. Meena S, Varla H, Swaminathan VV, et al. Hematopoietic stem cell Transplantation in Children with very Early Onset Inflammatory Bowel Disease Secondary to Monogenic Disorders of immune-dysregulation. *Indian J Hematol Blood Transfus*. Apr 2023;39(2):183–190. doi:10.1007/s12288-022-01586-2
84. Beşer Ö F, Conde CD, Kutlu T, Çullu Çokuğraş F, Boztuğ K, Erkan T. Inflammatory Bowel Disease With Lethal Disease Course Caused by a Nonsense Mutation in BIRC4 Encoding X-Linked Inhibitor of Apoptosis Protein (XIAP). *J Pediatr Gastroenterol Nutr*. May 2016;62(5):e41–3. doi:10.1097/mpg.0000000000000504
85. Otagiri S, Katsurada T, Sakurai K, Sugita J, Sakamoto N. Endoscopic balloon dilations for strictures of rectum, ileocecal valve and duodenum in a patient with X-linked inhibitor of apoptosis deficiency: a case report. *Intest Res*. Apr 2022;20(2):274–277. doi:10.5217/ir.2021.00029
86. Coelho R, Peixoto A, Amil-Dias J, et al. Refractory monogenic Crohn's disease due to X-linked inhibitor of apoptosis deficiency. *Int J Colorectal Dis*. Jun 2016;31(6):1235–6.

doi:10.1007/s00384-015-2442-0
87. Geerlinks AV, Dvorak AM. A Case of XIAP Deficiency Successfully Managed with Tadekinig Alfa (rhIL-18BP). *J Clin Immunol*. May 2022;42(4):901–903. doi:10.1007/s10875-022-01236-2
88. Mao L, Kitani A, Similuk M, et al. Loss-of-function CARD8 mutation causes NLRP3 inflammasome activation and Crohn's disease. *J Clin Invest*. May 1 2018;128(5):1793–1806. doi:10.1172/jci98642
89. Kotlarz D, Beier R, Murugan D, et al. Loss of interleukin-10 signaling and infantile inflammatory bowel disease: implications for diagnosis and therapy. *Gastroenterology*. Aug 2012;143(2):347–55. doi:10.1053/j.gastro.2012.04.045
90. Shouval DS, Konnikova L, Griffith AE, et al. Enhanced TH17 Responses in Patients with IL10 Receptor Deficiency and Infantile-onset IBD. *Inflamm Bowel Dis*. Nov 2017;23(11):1950–1961. doi:10.1097/mib.0000000000001270
91. Glocker EO, Frede N, Perro M, et al. Infant colitis--it's in the genes. *Lancet*. Oct 9 2010;376(9748):1272. doi:10.1016/s0140-6736(10)61008-2
92. Pigneur B, Escher J, Elawad M, et al. Phenotypic characterization of very early-onset IBD due to mutations in the IL10, IL10 receptor alpha or beta gene: a survey of the Genius Working Group. *Inflamm Bowel Dis*. Dec 2013;19(13):2820–8. doi:10.1097/01.MIB.0000435439.22484.d3
93. Engelhardt KR, Shah N, Faizura-Yeop I, et al. Clinical outcome in IL-10- and IL-10 receptor-deficient patients with or without hematopoietic stem cell transplantation. *J Allergy Clin Immunol*. Mar 2013;131(3):825–30. doi:10.1016/j.jaci.2012.09.025
94. Dinwiddie DL, Bracken JM, Bass JA, et al. Molecular diagnosis of infantile onset inflammatory bowel disease by exome sequencing. *Genomics*. Nov–Dec 2013;102(5-6):442–7. doi:10.1016/j.ygeno.2013.08.008
95. Murugan D, Albert MH, Langemeier J, et al. Very early onset inflammatory bowel disease associated with aberrant trafficking of IL-10R1 and cure by T cell replete haploidentical bone marrow transplantation. *J Clin Immunol*. Apr 2014;34(3):331–9. doi:10.1007/s10875-014-9992-8
96. McDonald BS, Narayanan S, Elawad M, Kiparissi F, Cullup T, Batta K. Interleukin-10 receptor mutation presenting with severe nappy ulceration and infantile inflammatory bowel disease. *Clin Exp Dermatol*. Oct 2017;42(7):771–773. doi:10.1111/ced.13157
97. Ye Z, Qian L, Hu W, et al. Clinical outcome of infantile-onset inflammatory bowel disease in 102 patients with interleukin-10 signalling deficiency. *Aliment Pharmacol Ther*. Jun 2022;55(11):1414–1422. doi:10.1111/apt.16837
98. Glocker EO, Kotlarz D, Boztug K, et al. Inflammatory bowel disease and mutations affecting the interleukin-10 receptor. *N Engl J Med*. Nov 19 2009;361(21):2033–45. doi:10.1056/NEJMoa0907206

99. Beser OF, Conde CD, Serwas NK, et al. Clinical features of interleukin 10 receptor gene mutations in children with very early-onset inflammatory bowel disease. *J Pediatr Gastroenterol Nutr*. Mar 2015;60(3):332–8. doi:10.1097/mpg.0000000000000621
100. Shouval DS, Ebens CL, Murchie R, et al. Large B-Cell Lymphoma in an Adolescent Patient With Interleukin-10 Receptor Deficiency and History of Infantile Inflammatory Bowel Disease. *J Pediatr Gastroenterol Nutr*. Jul 2016;63(1):e15–7. doi:10.1097/mpg.0000000000000532
101. Collen LV, Kim DY, Field M, et al. Clinical Phenotypes and Outcomes in Monogenic Versus Non-monogenic Very Early Onset Inflammatory Bowel Disease. *J Crohns Colitis*. Sep 8 2022;16(9):1380–1396. doi:10.1093/ecco-jcc/jjac045
102. Karaca NE, Aksu G, Ulusoy E, et al. Early Diagnosis and Hematopoietic Stem Cell Transplantation for IL10R Deficiency Leading to Very Early-Onset Inflammatory Bowel Disease Are Essential in Familial Cases. *Case Reports Immunol*. 2016;2016:5459029. doi:10.1155/2016/5459029
103. Ganesh R, Sathiyasekeran M, Srinivas S, Narayanan RK. Clinical Spectrum of Monogenic Infantile-Onset Inflammatory Bowel Disease. *Indian J Pediatr*. May 2022;89(5):497–502. doi:10.1007/s12098-022-04103-5
104. D'Agata ID, Paradis K, Chad Z, Bonny Y, Seidman E. Leucocyte adhesion deficiency presenting as a chronic ileocolitis. *Gut*. Oct 1996;39(4):605–8. doi:10.1136/gut.39.4.605
105. Van Eyck L, Jr., Hershfield MS, Pombal D, et al. Hematopoietic stem cell transplantation rescues the immunologic phenotype and prevents vasculopathy in patients with adenosine deaminase 2 deficiency. *J Allergy Clin Immunol*. Jan 2015;135(1):283–7.e5. doi:10.1016/j.jaci.2014.10.010
106. Hashem H, Kumar AR, Müller I, et al. Hematopoietic stem cell transplantation rescues the hematological, immunological, and vascular phenotype in DADA2. *Blood*. Dec 14 2017;130(24):2682–2688. doi:10.1182/blood-2017-07-798660
107. van Well GTJ, Kant B, van Nistelrooij A, et al. Phenotypic variability including Behçet's disease-like manifestations in DADA2 patients due to a homozygous c.973-2A>G splice site mutation. *Clin Exp Rheumatol*. Nov–Dec 2019;37 Suppl 121(6):142–146.
108. Lee PY, Kellner ES, Huang Y, et al. Genotype and functional correlates of disease phenotype in deficiency of adenosine deaminase 2 (DADA2). *J Allergy Clin Immunol*. Jun 2020;145(6):1664–1672.e10. doi:10.1016/j.jaci.2019.12.908
109. Süleyman M, Tan Ç, Uner A, et al. Adenosine Deaminase Type II Deficiency: Severe Chronic Neutropenia, Lymphoid Infiltration in Bone Marrow, and Inflammatory Features. *Immunol Invest*. Apr 2022;51(3):558–566. doi:10.1080/08820139.2020.1853153
110. Pimpale Chavan P, Ramadoss D, Khan A, Lee PY, Khubchandani R. Deficiency of Adenosine Deaminase 2 (DADA2): One Disease, Several Faces. *Indian J Pediatr*. Aug 2021;88(8):828–830. doi:10.1007/s12098-021-03809-2

111. Ouahed J, Kelsen JR, Spessott WA, et al. Variants in STXBP3 are Associated with Very Early Onset Inflammatory Bowel Disease, Bilateral Sensorineural Hearing Loss and Immune Dysregulation. *J Crohns Colitis*. Nov 8 2021;15(11):1908–1919. doi:10.1093/ecco-jcc/jjab077
112. Ozgür TT, Asal GT, Cetinkaya D, et al. Hematopoietic stem cell transplantation in a CD3 gamma-deficient infant with inflammatory bowel disease. *Pediatr Transplant*. Dec 2008;12(8):910–3. doi:10.1111/j.1399-3046.2008.00957.x
113. Ogawa A, Watanabe T, Natsume T, et al. Early-Onset Inflammatory Bowel Disease Caused by Mutations in the X-Linked Gene IL2RG. *J Investig Allergol Clin Immunol*. Feb 17 2021;31(1):69–71. doi:10.18176/jiaci.0523
114. Lin YF, Lee WI, Ho CH, et al. Lymphocyte disturbance and functional assessment of the [Asp521Asn] ZAP70 mutation. *Clin Immunol*. Feb 2023;247:109236. doi:10.1016/j.clim.2023.109236
115. Liu Q, Wang YP, Liu Q, et al. Novel compound heterozygous mutations in ZAP70 in a Chinese patient with leaky severe combined immunodeficiency disorder. *Immunogenetics*. Apr 2017;69(4):199–209. doi:10.1007/s00251-017-0971-0
116. Chan AY, Punwani D, Kadlecek TA, et al. A novel human autoimmune syndrome caused by combined hypomorphic and activating mutations in ZAP-70. *J Exp Med*. Feb 8 2016;213(2):155–65. doi:10.1084/jem.20150888
117. Rohr J, Pannicke U, Döring M, et al. Chronic inflammatory bowel disease as key manifestation of atypical ARTEMIS deficiency. *J Clin Immunol*. Mar 2010;30(2):314–20. doi:10.1007/s10875-009-9349-x
118. Khoury T, Molho-Pessach V, Ramot Y, et al. Tocilizumab Promotes Regulatory T-cell Alleviation in STAT3 Gain-of-function-associated Multi-organ Autoimmune Syndrome. *Clin Ther*. Feb 2017;39(2):444–449. doi:10.1016/j.clinthera.2017.01.004
119. Parlato M, Nian Q, Charbit-Henrion F, Ruemmele FM, Rodrigues-Lima F, Cerf-Bensussan N. Loss-of-Function Mutation in PTPN2 Causes Aberrant Activation of JAK Signaling Via STAT and Very Early Onset Intestinal Inflammation. *Gastroenterology*. Nov 2020;159(5):1968–1971.e4. doi:10.1053/j.gastro.2020.07.040
120. Zhang Z, Gothe F, Pennamen P, et al. Human interleukin-2 receptor β mutations associated with defects in immunity and peripheral tolerance. *J Exp Med*. Jun 3 2019;216(6):1311–1327. doi:10.1084/jem.20182304
121. Charbit-Henrion F, Jeverica AK, Bègue B, et al. Deficiency in Mucosa-associated Lymphoid Tissue Lymphoma Translocation 1: A Novel Cause of IPEX-Like Syndrome. *J Pediatr Gastroenterol Nutr*. Mar 2017;64(3):378–384. doi:10.1097/mpg.0000000000001262
122. Crowley E, Warner N, Pan J, et al. Prevalence and Clinical Features of Inflammatory Bowel Diseases Associated With Monogenic Variants, Identified by Whole-Exome Sequencing in 1000 Children at a Single Center. *Gastroenterology*. Jun 2020;158(8):2208–2220. doi:10.1053/j.gastro.2020.02.023

123. Kammermeier J, Dziubak R, Pescarin M, et al. Phenotypic and Genotypic Characterisation of Inflammatory Bowel Disease Presenting Before the Age of 2 years. *J Crohns Colitis*. Jan 2017;11(1):60–69. doi:10.1093/ecco-jcc/jjw118
124. Ge T, Wang Y, Che Y, Xiao Y, Zhang T. Atypical Late-Onset Immune Dysregulation, Polyendocrinopathy, Enteropathy, X-Linked Syndrome with Intractable Diarrhea: A Case Report. *Front Pediatr*. 2017;5:267. doi:10.3389/fped.2017.00267
125. Ke R, Zhu Y, Deng F, Xu D. Renal Involvement in IPEX Syndrome With a Novel Mutation of FOXP3: A Case Report. *Front Genet*. 2021;12:752775. doi:10.3389/fgene.2021.752775
126. Duclaux-Loras R, Charbit-Henrion F, Neven B, et al. Clinical Heterogeneity of Immune Dysregulation, Polyendocrinopathy, Enteropathy, X-Linked Syndrome: A French Multicenter Retrospective Study. *Clin Transl Gastroenterol*. Nov 2 2018;9(10):201. doi:10.1038/s41424-018-0064-x
127. Barzaghi F, Passerini L, Bacchetta R. Immune dysregulation, polyendocrinopathy, enteropathy, x-linked syndrome: a paradigm of immunodeficiency with autoimmunity. *Front Immunol*. 2012;3:211. doi:10.3389/fimmu.2012.00211
128. Yong PL, Russo P, Sullivan KE. Use of sirolimus in IPEX and IPEX-like children. *J Clin Immunol*. Sep 2008;28(5):581–7. doi:10.1007/s10875-008-9196-1
129. Lucas KG, Ungar D, Comito M, Bayerl M, Groh B. Submyeloablative cord blood transplantation corrects clinical defects seen in IPEX syndrome. *Bone Marrow Transplant*. Jan 2007;39(1):55–6. doi:10.1038/sj.bmt.1705542
130. Zeissig S, Petersen BS, Tomczak M, et al. Early-onset Crohn's disease and autoimmunity associated with a variant in CTLA-4. *Gut*. Dec 2015;64(12):1889–97. doi:10.1136/gutjnl-2014-308541
131. Constant BD, Dutmer CM, Arnold MA, Hall C, Abbott JK, de Zoeten EF. Cytotoxic T-Lymphocyte-Associated Antigen 4 Haploinsufficiency Mimics Difficult-to-Treat Inflammatory Bowel Disease. *Clin Gastroenterol Hepatol*. Apr 2022;20(4):e696–e702. doi:10.1016/j.cgh.2021.05.001
132. Schwab C, Gabrysch A, Olbrich P, et al. Phenotype, penetrance, and treatment of 133 cytotoxic T-lymphocyte antigen 4-insufficient subjects. *J Allergy Clin Immunol*. Dec 2018;142(6):1932–1946. doi:10.1016/j.jaci.2018.02.055
133. Tran NN, Setty M, Cham E, Chan AY, Ali S. CTLA-4 Haploinsufficiency Presenting as Extensive Enteropathy in a Patient With Very Early Onset Inflammatory Bowel Disease. *JPGN Rep*. Aug 2021;2(3):e099. doi:10.1097/pg9.0000000000000099
134. Slatter MA, Engelhardt KR, Burroughs LM, et al. Hematopoietic stem cell transplantation for CTLA4 deficiency. *J Allergy Clin Immunol*. Aug 2016;138(2):615–619.e1. doi:10.1016/j.jaci.2016.01.045
135. Kuehn HS, Ouyang W, Lo B, et al. Immune dysregulation in human subjects with heterozygous germline mutations in CTLA4. *Science*. Sep 26 2014;345(6204):1623–1627.

doi:10.1126/science.1255904
136. Egg D, Rump IC, Mitsuiki N, et al. Therapeutic options for CTLA-4 insufficiency. *J Allergy Clin Immunol*. Feb 2022;149(2):736–746. doi:10.1016/j.jaci.2021.04.039
137. Navarini AA, Hruz P, Berger CT, et al. Vedolizumab as a successful treatment of CTLA-4-associated autoimmune enterocolitis. *J Allergy Clin Immunol*. Mar 2017;139(3):1043–1046.e5. doi:10.1016/j.jaci.2016.08.042
138. Serwas NK, Kansu A, Santos-Valente E, et al. Atypical manifestation of LRBA deficiency with predominant IBD-like phenotype. *Inflamm Bowel Dis*. Jan 2015;21(1):40–7. doi:10.1097/mib.0000000000000266
139. Kostel Bal S, Haskologlu S, Serwas NK, et al. Multiple Presentations of LRBA Deficiency: a Single-Center Experience. *J Clin Immunol*. Nov 2017;37(8):790–800. doi:10.1007/s10875-017-0446-y
140. Lo B, Zhang K, Lu W, et al. AUTOIMMUNE DISEASE. Patients with LRBA deficiency show CTLA4 loss and immune dysregulation responsive to abatacept therapy. *Science*. Jul 24 2015;349(6246):436–40. doi:10.1126/science.aaa1663
141. Banday AZ, Jindal AK, Tyagi R, et al. Refractory Autoimmune Cytopenia in a Young Boy with a Novel LRBA Mutation Successfully Managed with Sirolimus. *J Clin Immunol*. Nov 2020;40(8):1184–1186. doi:10.1007/s10875-020-00835-1
142. He M, Wong A, Sutton K, Gondim MJB, Samson C. Very-Early Onset Chronic Active Colitis with Heterozygous Variants in LRBA1 and CARD11, a Case of "Immune TOR-Opathies". *Fetal Pediatr Pathol*. Apr 2023;42(2):297–306. doi:10.1080/15513815.2022.2088912
143. Fabozzi F, De Vito R, Gaspari S, et al. Case report: A new pathogenic variant of LRBA deficiency with a complex phenotype and Rosai-Dorfman disease. *Front Immunol*. 2022;13:944810. doi:10.3389/fimmu.2022.944810
144. Azizi G, Abolhassani H, Yazdani R, et al. New therapeutic approach by sirolimus for enteropathy treatment in patients with LRBA deficiency. *Eur Ann Allergy Clin Immunol*. Sep 2017;49(5):235–239. doi:10.23822/EurAnnACI.1764-1489.22
145. Miller TL, Lee D, Giefer M, Wahbeh G, Suskind DL. Nutritional Therapy in Very Early-Onset Inflammatory Bowel Disease: A Case Report. *Dig Dis Sci*. Aug 2017;62(8):2196–2200. doi:10.1007/s10620-017-4616-9
146. Bakhtiar S, Gámez-Díaz L, Jarisch A, et al. Treatment of Infantile Inflammatory Bowel Disease and Autoimmunity by Allogeneic Stem Cell Transplantation in LPS-Responsive Beige-Like Anchor Deficiency. *Front Immunol*. 2017;8:52. doi:10.3389/fimmu.2017.00052
147. Ye Z, Zhou Y, Huang Y, et al. Phenotype and Management of Infantile-onset Inflammatory Bowel Disease: Experience from a Tertiary Care Center in China. *Inflamm Bowel Dis*. Dec 2017;23(12):2154–2164. doi:10.1097/mib.0000000000001269
148. Tang WJ, Hu WH, Huang Y, et al. Potential protein-phenotype correlation in three lipopolysaccharide-responsive beige-like anchor protein-deficient patients. *World J Clin*

Cases. Jul 26 2021;9(21):5873–5888. doi:10.12998/wjcc.v9.i21.5873
149. Schreiner F, Plamper M, Dueker G, et al. Infancy-Onset T1DM, Short Stature, and Severe Immunodysregulation in Two Siblings With a Homozygous LRBA Mutation. *J Clin Endocrinol Metab*. Mar 2016;101(3):898–904. doi:10.1210/jc.2015-3382
150. Tesch VK, Abolhassani H, Shadur B, et al. Long-term outcome of LRBA deficiency in 76 patients after various treatment modalities as evaluated by the immune deficiency and dysregulation activity (IDDA) score. *J Allergy Clin Immunol*. May 2020;145(5):1452–1463. doi:10.1016/j.jaci.2019.12.896
151. Günaydin NC, Chou J, Karaca NE, et al. A novel disease-causing CD40L mutation reduces expression of CD40 ligand, but preserves CD40 binding capacity. *Clin Immunol*. Aug 2014;153(2):288–91. doi:10.1016/j.clim.2014.05.001
152. Qiu KY, Liao XY, Wu RH, Huang K, Fang JP, Zhou DH. X-linked Hyper-IgM Syndrome: A Phenotype of Crohn's Disease with Hemophagocytic Lymphohistiocytosis. *Pediatr Hematol Oncol*. Nov 2017;34(8):428–434. doi:10.1080/08880018.2017.1409301
153. Shamriz O, Simon AJ, Lev A, et al. Exogenous interleukin-2 can rescue in-vitro T cell activation and proliferation in patients with a novel capping protein regulator and myosin 1 linker 2 mutation. *Clin Exp Immunol*. Jun 2020;200(3):215–227. doi:10.1111/cei.13432
154. Kolukisa B, Baser D, Akcam B, et al. Evolution and long-term outcomes of combined immunodeficiency due to CARMIL2 deficiency. *Allergy*. Mar 2022;77(3):1004–1019. doi:10.1111/all.15010
155. Bosa L, Batura V, Colavito D, et al. Novel CARMIL2 loss-of-function variants are associated with pediatric inflammatory bowel disease. *Sci Rep*. Mar 15 2021;11(1):5945. doi:10.1038/s41598-021-85399-9
156. Magg T, Shcherbina A, Arslan D, et al. CARMIL2 Deficiency Presenting as Very Early Onset Inflammatory Bowel Disease. *Inflamm Bowel Dis*. Oct 18 2019;25(11):1788–1795. doi:10.1093/ibd/izz103
157. Huang H, Yang X, Tao L, Xiang R, Yang H. Identification of a de novo heterozygous mutation of ANKZF1 in a Chinese patient with inflammatory bowel disease. *Qjm*. Jun 8 2023;116(6):463–465. doi:10.1093/qjmed/hcad030
158. Takahashi N, Matsumoto K, Saito H, et al. Impaired CD4 and CD8 effector function and decreased memory T cell populations in ICOS-deficient patients. *J Immunol*. May 1 2009;182(9):5515–27. doi:10.4049/jimmunol.0803256
159. Sifuentes-Dominguez LF, Li H, Llano E, et al. SCGN deficiency results in colitis susceptibility. *Elife*. Oct 30 2019;8doi:10.7554/eLife.49910
160. Sun G, Wu M, Lv Q, et al. A Multicenter Cohort Study of Immune Dysregulation Disorders Caused by ELF4 Variants in China. *J Clin Immunol*. Jul 2023;43(5):933–939. doi:10.1007/s10875-023-01453-3
161. Chen YE, Chen J, Guo W, et al. Clinical Characteristics, In Silico Analysis, and Intervention

of Neonatal-Onset Inflammatory Bowel Disease With Combined Immunodeficiency Caused by Novel TTC7A Variants. *Front Genet*. 2022;13:921808. doi:10.3389/fgene.2022.921808
162. Diociaiuti A, Caruso R, Ricci S, et al. Prominent Follicular Keratosis in Multiple Intestinal Atresia with Combined Immune Deficiency Caused by a TTC7A Homozygous Mutation. *Genes (Basel)*. May 4 2022;13(5)doi:10.3390/genes13050821
163. Lien R, Lin YF, Lai MW, et al. Novel Mutations of the Tetratricopeptide Repeat Domain 7A Gene and Phenotype/Genotype Comparison. *Front Immunol*. 2017;8:1066. doi:10.3389/fimmu.2017.01066
164. Broome DT, Young A, Torbic H, et al. Combined Immunodeficiency With Inflammatory Bowel Disease in a Patient With TTC7A Deficiency. *ACG Case Rep J*. May 2019;6(5):e00061. doi:10.14309/crj.0000000000000061
165. Mandiá N, Pérez-Muñuzuri A, López-Suárez O, López-Sanguos C, Bautista-Casanovas A, Couce ML. Congenital intestinal atresias with multiple episodes of sepsis: A case report and review of literature. *Medicine (Baltimore)*. Jun 2018;97(23):e10939. doi:10.1097/md.0000000000010939
166. Kammermeier J, Lucchini G, Pai SY, et al. Stem cell transplantation for tetratricopeptide repeat domain 7A deficiency: long-term follow-up. *Blood*. Sep 1 2016;128(9):1306–8. doi:10.1182/blood-2016-01-696385
167. Fernandez I, Patey N, Marchand V, et al. Multiple intestinal atresia with combined immune deficiency related to TTC7A defect is a multiorgan pathology: study of a French-Canadian-based cohort. *Medicine (Baltimore)*. Dec 2014;93(29):e327. doi:10.1097/md.0000000000000327
168. Chen R, Giliani S, Lanzi G, et al. Whole-exome sequencing identifies tetratricopeptide repeat domain 7A (TTC7A) mutations for combined immunodeficiency with intestinal atresias. *J Allergy Clin Immunol*. Sep 2013;132(3):656–664.e17. doi:10.1016/j.jaci.2013.06.013
169. O'Connell AE, Zhou F, Shah MS, et al. Neonatal-Onset Chronic Diarrhea Caused by Homozygous Nonsense WNT2B Mutations. *Am J Hum Genet*. Jul 5 2018;103(1):131–137. doi:10.1016/j.ajhg.2018.05.007
170. Norsa L, Berni Canani R, Duclaux-Loras R, et al. Inflammatory Bowel Disease in Patients with Congenital Chloride Diarrhoea. *J Crohns Colitis*. Oct 7 2021;15(10):1679–1685. doi:10.1093/ecco-jcc/jjab056
171. Kammermeier J, Drury S, James CT, et al. Targeted gene panel sequencing in children with very early onset inflammatory bowel disease--evaluation and prospective analysis. *J Med Genet*. Nov 2014;51(11):748–55. doi:10.1136/jmedgenet-2014-102624
172. Busoni VB, Lemale J, Dubern B, et al. IBD-Like Features in Syndromic Diarrhea/Trichohepatoenteric Syndrome. *J Pediatr Gastroenterol Nutr*. Jan 2017;64(1):37–41. doi:10.1097/mpg.0000000000001218

173. Lee WI, Huang JL, Chen CC, et al. Identifying Mutations of the Tetratricopeptide Repeat Domain 37 (TTC37) Gene in Infants With Intractable Diarrhea and a Comparison of Asian and Non-Asian Phenotype and Genotype: A Global Case-report Study of a Well-Defined Syndrome With Immunodeficiency. *Medicine (Baltimore)*. Mar 2016;95(9):e2918. doi:10.1097/md.0000000000002918
174. Talbot J, Wiskin AE, Monaghan M, Goenka A, Rice G, Roderick M. Management of Enteritis Associated With Tricohepatoenteric Syndrome due to SKIV2L Mutation Using the Combination of JAK1/2 Inhibition and Azathioprine. *JPGN Rep*. Nov 2022;3(4):e264. doi:10.1097/pg9.0000000000000264
175. Shimizu S, Nishie W, Tsuchiya K, et al. Kindler syndrome with severe intestinal involvement: a 31-year follow-up. *Acta Derm Venereol*. May 2014;94(3):351–2. doi:10.2340/00015555-1696
176. Parlato M, Charbit-Henrion F, Pan J, et al. Human ALPI deficiency causes inflammatory bowel disease and highlights a key mechanism of gut homeostasis. *EMBO Mol Med*. Apr 2018;10(4)doi:10.15252/emmm.201708483
177. Sultan M, Adawi M, Kol N, et al. RIPK1 mutations causing infantile-onset IBD with inflammatory and fistulizing features. *Front Immunol*. 2022;13:1041315. doi:10.3389/fimmu.2022.1041315
178. Li Y, Führer M, Bahrami E, et al. Human RIPK1 deficiency causes combined immunodeficiency and inflammatory bowel diseases. *Proc Natl Acad Sci U S A*. Jan 15 2019;116(3):970–975. doi:10.1073/pnas.1813582116
179. Lin L, Wang Y, Liu L, et al. Clinical phenotype of a Chinese patient with RIPK1 deficiency due to novel mutation. *Genes Dis*. Mar 2020;7(1):122–127. doi:10.1016/j.gendis.2019.10.008
180. Lehle AS, Farin HF, Marquardt B, et al. Intestinal Inflammation and Dysregulated Immunity in Patients With Inherited Caspase-8 Deficiency. *Gastroenterology*. Jan 2019;156(1):275–278. doi:10.1053/j.gastro.2018.09.041
181. Bazgir N, Tahvildari A, Chavoshzade Z, et al. A rare immunological disease, caspase 8 deficiency: case report and literature review. *Allergy Asthma Clin Immunol*. Apr 10 2023;19(1):29. doi:10.1186/s13223-023-00778-3
182. Boisson B, Laplantine E, Prando C, et al. Immunodeficiency, autoinflammation and amylopectinosis in humans with inherited HOIL-1 and LUBAC deficiency. *Nat Immunol*. Dec 2012;13(12):1178–86. doi:10.1038/ni.2457
183. Shiraki M, Williams E, Yokoyama N, et al. Hematopoietic Cell Transplantation Ameliorates Autoinflammation in A20 Haploinsufficiency. *J Clin Immunol*. Nov 2021;41(8):1954–1956. doi:10.1007/s10875-021-01124-1
184. Kadowaki T, Ohnishi H, Kawamoto N, et al. Haploinsufficiency of A20 causes autoinflammatory and autoimmune disorders. *J Allergy Clin Immunol*. Apr

2018;141(4):1485–1488.e11. doi:10.1016/j.jaci.2017.10.039
185. Aeschlimann FA, Batu ED, Canna SW, et al. A20 haploinsufficiency (HA20): clinical phenotypes and disease course of patients with a newly recognised NF-kB-mediated autoinflammatory disease. *Ann Rheum Dis*. May 2018;77(5):728–735. doi:10.1136/annrheumdis-2017-212403
186. Zhang D, Su G, Zhou Z, Lai J. Clinical characteristics and genetic analysis of A20 haploinsufficiency. *Pediatr Rheumatol Online J*. May 24 2021;19(1):75. doi:10.1186/s12969-021-00558-6
187. Chen Y, Huang H, He Y, et al. A20 Haploinsufficiency in a Chinese Patient With Intestinal Behcet's Disease-Like Symptoms: A Case Report. *Front Immunol*. 2020;11:1414. doi:10.3389/fimmu.2020.01414
188. He T, Huang Y, Luo Y, et al. Haploinsufficiency of A20 Due to Novel Mutations in TNFAIP3. *J Clin Immunol*. Jul 2020;40(5):741–751. doi:10.1007/s10875-020-00792-9
189. Schwartz DM, Blackstone SA, Sampaio-Moura N, et al. Type I interferon signature predicts response to JAK inhibition in haploinsufficiency of A20. *Ann Rheum Dis*. Mar 2020;79(3):429–431. doi:10.1136/annrheumdis-2019-215918
190. Wakatsuki R, Hatai Y, Okamoto K, et al. An infant with A20 haploinsufficiency presenting with periodic fever syndrome: A case report. *Int J Rheum Dis*. May 2023;26(5):973–976. doi:10.1111/1756-185x.14564
191. Zanatta L, Biscaro F, Bresolin S, et al. Case Report: An early-onset inflammatory colitis due to a variant in TNFAIP3 causing A20 haploinsufficiency. *Front Pediatr*. 2022;10:1044007. doi:10.3389/fped.2022.1044007
192. Jiang W, Deng M, Gan C, Wang L, Mao H, Li Q. A novel missense mutation in TNFAIP3 causes haploinsufficiency of A20. *Cell Immunol*. Jan 2022;371:104453. doi:10.1016/j.cellimm.2021.104453
193. Kadowaki S, Hashimoto K, Nishimura T, et al. Functional analysis of novel A20 variants in patients with atypical inflammatory diseases. *Arthritis Res Ther*. Feb 6 2021;23(1):52. doi:10.1186/s13075-021-02434-w
194. Taniguchi K, Inoue M, Arai K, et al. Novel TNFAIP3 microdeletion in a girl with infantile-onset inflammatory bowel disease complicated by a severe perianal lesion. *Hum Genome Var*. Jan 14 2021;8(1):1. doi:10.1038/s41439-020-00128-4
195. Li GM, Liu HM, Guan WZ, Xu H, Wu BB, Sun L. Expanding the spectrum of A20 haploinsufficiency in two Chinese families: cases report. *BMC Med Genet*. Jul 12 2019;20(1):124. doi:10.1186/s12881-019-0856-1
196. Tsuchida N, Kirino Y, Soejima Y, et al. Haploinsufficiency of A20 caused by a novel nonsense variant or entire deletion of TNFAIP3 is clinically distinct from Behçet's disease. *Arthritis Res Ther*. Jun 4 2019;21(1):137. doi:10.1186/s13075-019-1928-5
197. Shimizu M, Matsubayashi T, Ohnishi H, et al. Haploinsufficiency of A20 with a novel

mutation of deletion of exons 2-3 of TNFAIP3. *Mod Rheumatol*. Mar 2021;31(2):493–497. doi:10.1080/14397595.2020.1719595
198. Hu W, Ye Z, Li P, Wang Y, Huang Y. Very Early Onset Inflammatory Bowel Disease Caused by a Novel Dominant Negative Mutation of Caspase Recruitment Domain 11 (CARD11). *J Clin Immunol*. Apr 2023;43(3):528–531. doi:10.1007/s10875-022-01387-2
199. Lu HY, Sharma M, Sharma AA, et al. Mechanistic understanding of the combined immunodeficiency in complete human CARD11 deficiency. *J Allergy Clin Immunol*. Dec 2021;148(6):1559–1574.e13. doi:10.1016/j.jaci.2021.04.006
200. Badran YR, Dedeoglu F, Leyva Castillo JM, et al. Human RELA haploinsufficiency results in autosomal-dominant chronic mucocutaneous ulceration. *J Exp Med*. Jul 3 2017;214(7):1937–1947. doi:10.1084/jem.20160724
201. Uchida T, Suzuki T, Kikuchi A, et al. Comprehensive Targeted Sequencing Identifies Monogenic Disorders in Patients With Early-onset Refractory Diarrhea. *J Pediatr Gastroenterol Nutr*. Sep 2020;71(3):333–339. doi:10.1097/mpg.0000000000002796
202. Schimke LF, Rieber N, Rylaarsdam S, et al. A novel gain-of-function IKBA mutation underlies ectodermal dysplasia with immunodeficiency and polyendocrinopathy. *J Clin Immunol*. Aug 2013;33(6):1088–99. doi:10.1007/s10875-013-9906-1
203. Courtois G, Smahi A, Reichenbach J, et al. A hypermorphic IkappaBalpha mutation is associated with autosomal dominant anhidrotic ectodermal dysplasia and T cell immunodeficiency. *J Clin Invest*. Oct 2003;112(7):1108–15. doi:10.1172/jci18714
204. Tegtmeyer D, Seidl M, Gerner P, Baumann U, Klemann C. Inflammatory bowel disease caused by primary immunodeficiencies-Clinical presentations, review of literature, and proposal of a rational diagnostic algorithm. *Pediatr Allergy Immunol*. Aug 2017;28(5):412–429. doi:10.1111/pai.12734
205. Mizukami T, Obara M, Nishikomori R, et al. Successful treatment with infliximab for inflammatory colitis in a patient with X-linked anhidrotic ectodermal dysplasia with immunodeficiency. *J Clin Immunol*. Feb 2012;32(1):39–49. doi:10.1007/s10875-011-9600-0
206. Artac H, Emsen A, Ucaryilmaz H, Emiroglu HH, Uygun V, Stray-Pedersen A. Infliximab therapy for inflammatory colitis in an infant with NEMO deficiency. *Immunol Res*. Oct 2019;67(4-5):450–453. doi:10.1007/s12026-019-09100-z
207. Miot C, Imai K, Imai C, et al. Hematopoietic stem cell transplantation in 29 patients hemizygous for hypomorphic IKBKG/NEMO mutations. *Blood*. Sep 21 2017;130(12):1456–1467. doi:10.1182/blood-2017-03-771600
208. Ishihara J, Mizuochi T, Uchida T, et al. Infantile-onset inflammatory bowel disease in a patient with Hermansky-Pudlak syndrome: a case report. *BMC Gastroenterol*. Jan 11 2019;19(1):9. doi:10.1186/s12876-019-0929-9
209. Salvaggio HL, Graeber KE, Clarke LE, Schlosser BJ, Orlow SJ, Clarke JT. Mucocutaneous granulomatous disease in a patient with Hermansky-Pudlak syndrome. *JAMA Dermatol*. Oct

2014;150(10):1083–7. doi:10.1001/jamadermatol.2014.147
210. Girot P, Le Berre C, De Maissin A, Freyssinet M, Trang-Poisson C, Bourreille A. Crohn's-like acute severe colitis associated with Hermansky-Pudlak syndrome: A case report. *World J Gastroenterol*. Feb 28 2019;25(8):1031–1036. doi:10.3748/wjg.v25.i8.1031
211. Felipez LM, Gokhale R, Guandalini S. Hermansky-Pudlak syndrome: severe colitis and good response to infliximab. *J Pediatr Gastroenterol Nutr*. Nov 2010;51(5):665–7. doi:10.1097/MPG.0b013e3181d2dcbb
212. Lozynska LY, Plawski A, Lozynska MR, et al. Variant of rare Hermansky - Pudlak syndrome associated with granulomatous colitis: diagnostics, clinical course and treatment. *Exp Oncol*. Mar 2018;40(1):73–78.
213. Moriya K, Niizuma H, Rikiishi T, Yamaguchi H, Sasahara Y, Kure S. Novel Compound Heterozygous RTEL1 Gene Mutations in a Patient With Hoyeraal-Hreidarsson Syndrome. *Pediatr Blood Cancer*. Sep 2016;63(9):1683–4. doi:10.1002/pbc.26030
214. Kotlarz D, Marquardt B, Barøy T, et al. Human TGF-β1 deficiency causes severe inflammatory bowel disease and encephalopathy. *Nat Genet*. Mar 2018;50(3):344–348. doi:10.1038/s41588-018-0063-6
215. Guerrerio AL, Frischmeyer-Guerrerio PA, Huang C, et al. Increased Prevalence of Inflammatory Bowel Disease in Patients with Mutations in Genes Encoding the Receptor Subunits for TGFβ. *Inflamm Bowel Dis*. Sep 2016;22(9):2058–2062. doi:10.1097/mib.0000000000000872
216. Naviglio S, Arrigo S, Martelossi S, et al. Severe inflammatory bowel disease associated with congenital alteration of transforming growth factor beta signaling. *J Crohns Colitis*. Aug 2014;8(8):770–4. doi:10.1016/j.crohns.2014.01.013
217. Opréa A, Collardeau-Frachon S, Heissat S, Peretti N, Lachaux A, Duclaux-Loras R. Therapeutic Approach of Very Early-Onset Inflammatory Bowel Disease in a Loeys-Dietz Syndrome Child. *JPGN Rep*. Feb 2022;3(1):e139. doi:10.1097/pg9.0000000000000139
218. Canna SW, Girard C, Malle L, et al. Life-threatening NLRC4-associated hyperinflammation successfully treated with IL-18 inhibition. *J Allergy Clin Immunol*. May 2017;139(5):1698–1701. doi:10.1016/j.jaci.2016.10.022
219. Steiner A, Reygaerts T, Pontillo A, et al. Recessive NLRC4-Autoinflammatory Disease Reveals an Ulcerative Colitis Locus. *J Clin Immunol*. Feb 2022;42(2):325–335. doi:10.1007/s10875-021-01175-4
220. Lohr NJ, Molleston JP, Strauss KA, et al. Human ITCH E3 ubiquitin ligase deficiency causes syndromic multisystem autoimmune disease. *Am J Hum Genet*. Mar 12 2010;86(3):447–53. doi:10.1016/j.ajhg.2010.01.028
221. Wolfe R, Heiman P, D'Annibale O, et al. ITCH deficiency clinical phenotype expansion and mitochondrial dysfunction. *Mol Genet Metab Rep*. Dec 2022;33:100932. doi:10.1016/j.ymgmr.2022.100932

222. Patel T, Henrickson SE, Moser EK, et al. Immune Dysregulation in Human ITCH Deficiency Successfully Treated with Hematopoietic Cell Transplantation. *J Allergy Clin Immunol Pract*. Jul 2021;9(7):2885–2893.e3. doi:10.1016/j.jaip.2021.04.010
223. Bader-Meunier B, Martins AL, Charbit-Henrion F, et al. Mevalonate Kinase Deficiency: A Cause of Severe Very-Early-Onset Inflammatory Bowel Disease. *Inflamm Bowel Dis*. Oct 20 2021;27(11):1853–1857. doi:10.1093/ibd/izab139
224. Bader-Meunier B, Florkin B, Sibilia J, et al. Mevalonate kinase deficiency: a survey of 50 patients. *Pediatrics*. Jul 2011;128(1):e152–9. doi:10.1542/peds.2010-3639
225. Miray Kisla Ekinci R, Balcı S, Bisgin A, Tumgor G, Dogruel D, Yilmaz M. Hyperimmunoglobulinemia D syndrome with recurrent perianal abscess successfully treated with canakinumab. *Scott Med J*. Aug 2019;64(3):103–107. doi:10.1177/0036933019836405
226. Levy M, Arion A, Berrebi D, et al. Severe early-onset colitis revealing mevalonate kinase deficiency. *Pediatrics*. Sep 2013;132(3):e779–83. doi:10.1542/peds.2012-3344
227. Ozen A, Comrie WA, Ardy RC, et al. CD55 Deficiency, Early-Onset Protein-Losing Enteropathy, and Thrombosis. *N Engl J Med*. Jul 6 2017;377(1):52–61. doi:10.1056/NEJMoa1615887
228. Ozen A, Kasap N, Vujkovic-Cvijin I, et al. Broadly effective metabolic and immune recovery with C5 inhibition in CHAPLE disease. *Nat Immunol*. Feb 2021;22(2):128–139. doi:10.1038/s41590-020-00830-z
229. Kurolap A, Eshach-Adiv O, Hershkovitz T, et al. Loss of CD55 in Eculizumab-Responsive Protein-Losing Enteropathy. *N Engl J Med*. Jul 6 2017;377(1):87–89. doi:10.1056/NEJMc1707173
230. Kurolap A, Eshach Adiv O, Hershkovitz T, et al. Eculizumab Is Safe and Effective as a Long-term Treatment for Protein-losing Enteropathy Due to CD55 Deficiency. *J Pediatr Gastroenterol Nutr*. Mar 2019;68(3):325–333. doi:10.1097/mpg.0000000000002198
231. Kurolap A, Hagin D, Freund T, et al. CD55-deficiency in Jews of Bukharan descent is caused by the Cromer blood type Dr(a-) variant. *Hum Genet*. May 2023;142(5):683–690. doi:10.1007/s00439-021-02428-3
232. Hagin D, Lahav D, Freund T, et al. Eculizumab-Responsive Adult Onset Protein Losing Enteropathy, Caused by Germline CD55-Deficiency and Complicated by Aggressive Angiosarcoma. *J Clin Immunol*. Feb 2021;41(2):477–481. doi:10.1007/s10875-020-00910-7
233. Takahashi S, Andreoletti G, Chen R, et al. De novo and rare mutations in the HSPA1L heat shock gene associated with inflammatory bowel disease. *Genome Med*. Jan 26 2017;9(1):8. doi:10.1186/s13073-016-0394-9
234. Hong HS, Baek J, Park JC, et al. Clinical and Genetic Characteristics of Korean Patients Diagnosed with Chronic Enteropathy Associated with SLCO2A1 Gene: A KASID Multicenter Study. *Gut Liver*. Nov 15 2022;16(6):942–951. doi:10.5009/gnl210415
235. Hamon A, Cazals-Hatem D, Stefanescu C, et al. Crohn-like Disease Affecting Small Bowel

Due to Monogenic SLCO2A1 Mutations: First Cases of Chronic Enteropathy Associated with SLCO2A1 Gene [CEAS] in France. *J Crohns Colitis*. May 3 2023;17(5):816–820. doi:10.1093/ecco-jcc/jjac181
236. Wang Q, Li YH, Lin GL, et al. Primary hypertrophic osteoarthropathy related gastrointestinal complication has distinctive clinical and pathological characteristics: two cases report and review of the literature. *Orphanet J Rare Dis*. Dec 26 2019;14(1):297. doi:10.1186/s13023-019-1264-5
237. Giannelou A, Wang H, Zhou Q, et al. Aberrant tRNA processing causes an autoinflammatory syndrome responsive to TNF inhibitors. *Ann Rheum Dis*. Apr 2018;77(4):612–619. doi:10.1136/annrheumdis-2017-212401
238. Frans G, Moens L, Schaballie H, et al. Homozygous N-terminal missense mutation in TRNT1 leads to progressive B-cell immunodeficiency in adulthood. *J Allergy Clin Immunol*. Jan 2017;139(1):360–363.e6. doi:10.1016/j.jaci.2016.06.050
239. Chen X, Fu F, Mo X, Cheng S, Zeng H. Case report: Sideroblastic anemia with B-cell immunodeficiency, periodic fevers, and developmental delay: Three cases and a literature review. *Front Pediatr*. 2023;11:1001222. doi:10.3389/fped.2023.1001222
240. Pan Y, Shang G, Li J, et al. Case Report: A novel IRF2BP2 mutation in an IEI patient with recurrent infections and autoimmune disorders. *Front Immunol*. 2023;14:967345. doi:10.3389/fimmu.2023.967345
241. Klemann C, Camacho-Ordonez N, Yang L, et al. Clinical and Immunological Phenotype of Patients With Primary Immunodeficiency Due to Damaging Mutations in NFKB2. *Front Immunol*. 2019;10:297. doi:10.3389/fimmu.2019.00297
